# Emergency communications after earthquake reveal social network backbone of important ties

**DOI:** 10.1093/pnasnexus/pgad358

**Published:** 2023-11-02

**Authors:** Jayson S Jia, Yiwei Li, Sheng Liu, Nicholas A Christakis, Jianmin Jia

**Affiliations:** Faculty of Business and Economics, The University of Hong Kong, Hong Kong SAR, China; Department of Marketing & International Business, Faculty of Business, Lingnan University, Hong Kong SAR, China; Department of Marketing & International Business, Faculty of Business, Lingnan University, Hong Kong SAR, China; Yale Institute for Network Science, Yale University, New Haven, CT 06520, USA; Shenzhen Finance Institute, School of Management and Economics, The Chinese University of Hong Kong, Shenzhen, China; Shenzhen Institute of Artificial Intelligence and Robotics for Society, Shenzhen, China

**Keywords:** tie strength, structural embeddedness, social network activation, quasi-experiment, earthquake disaster

## Abstract

Social networks provide a basis for collective resilience to disasters. Combining the quasi-experimental context of a major earthquake in Ya’an, China, with anonymized mobile telecommunications records regarding 91,839 Ya’an residents, we use initial bursts of postdisaster communications (e.g. choice of alter, order of calls, and latency) to reveal the “important ties” that form the social network backbone. We find that only 26.8% of important ties activated during the earthquake were the strongest ties during normal times. Many important ties were hitherto latent and weak, only to become persistent and strong after the earthquake. We show that which ties activated during a sudden disaster are best predicted by the interaction of embeddedness and tie strength. Moreover, a backbone of important ties alone (without the inclusion of weak ties ordinarily seen as important to bridge communities) is sufficient to generate a hierarchical structure of social networks that connect a disaster zone's disparate communities.

**Significance Statement**

In exploring the social network dynamics after a sudden natural disaster, we use the logic of revealed (social) preference (based on real communications decisions during emergency) to reveal which ties were most important and resilient. We find that tie strength, the most common measure for relationship strength, has limited ability to predict which social ties are prioritized: tie strength predicts tie importance for embedded ties but not necessarily for unembedded ties. These findings challenge common assumptions of empirical social networks research, where frequency-based tie strength and embeddedness measures are often used as independent measures of relationship strength. Rather, a joint assessment of both the dyadic relationship's historical strength and its structural embeddedness with the wider social network is necessary to reveal how reliable and resilient social ties are during emergencies.

## Introduction

The long-term integrity of human social systems depends in part on their ability to weather disasters, which have continuously afflicted societies throughout recorded history and which continue to loom large under the specters of climate change, population pressures, and ecological destruction (1–3). During disasters, social networks provide a decentralized basis for society's collective resilience. Social networks can help households mobilize resources (4–6), provide psychological support (7, 8), and access information (e.g. coping strategies) and can even compensate for the lack of human capital (9). Recent empirical research in the emerging field of computational social science has shed light on how disasters affect social media usage, economic transfers, and migration patterns (6, 7, 10–15). However, there is little empirical understanding, particularly at the population level, of how social network dynamics might change during emergencies, for example, who victims first turn to for support, who comprises the backbone network that activates during emergency, or the macrofeatures of this emergent social network.

Here, we explore such questions and also attempt to shed light on the theoretical basis of relationship strength, which provides the microlevel foundation for social interconnectivity and resilience during disaster. We combine the quasi-experimental context of the 2013 Ya’an earthquake (M_s_ 7.0) in Sichuan, China, with anonymized mobile phone records (CDR, Call Detail Record) of 91,839 residents of Ya’an and analyze how communications unfolded immediately before and after the prefecture was stricken. We use these uncommon data to identify who earthquake victims prioritized after the earthquake. For example, we sought to predict who victims called first, social ties we refer to as “important ties.” This behavioral measure of “revealed tie importance” (e.g. choice of alter, order of calls, and communications urgency or latency) is analogous to the axiomatic concept of revealed preference in economics, namely, that our observed choice between objects in a set reveals our relative preferences for those objects (16). In the immediate aftermath of disaster, victims may activate social networks to share information about their wellbeing, plan future action, request assistance, and seek emotional support. In this context, who victims first call (and the urgency and latency of their communications) is a meaningful signal of relationship importance and reveals a ground truth that is difficult to observe under normal circumstances.

## Conceptual background

Tie strength was originally defined as a reflection of the “the emotional intensity, the intimacy, and the reciprocal services which characterize a [social] tie” (17). Extant empirical research generally assumes that tie strength and the quality of a relationship can be measured by the frequency (i.e. “intensity”) of interactions (18–21). In recent empirical literature, tie strength has been used to operationalize relationship closeness, depth, and even emotional connection (18–26). This perspective suggests that an ego's most frequently contacted alters are their strongest and most important relationships.

We investigate and challenge this assumption by using observed choices (ranking or temporal sequence of who earthquake victims first call) during an earthquake to infer relationship importance, which we then compare to relationship strength measures, such as (preearthquake) tie strength. We later show that tie strength, by itself, is a weak predictor of tie importance and social network activation behavior immediately after the earthquake.

We hypothesize that the limited predictive power of tie strength may be due to its dyadic nature, which, by virtue of ignoring the structural aspect of social relationships, offers an incomplete assessment of what makes relationships deep and resilient. Indeed, the classic “strength of weak ties” concept also theorizes that “all social action and outcomes, are affected by actors’ dyadic (pair-wise) relations and by the structure of the overall network of relations” (27). In other words, whether people are willing to engage in social action for each other depends not only on (i) their pair-wise relationship strength but also on (ii) how they are *embedded* within the wider social network (17, 27). The former construct is often called “relational embeddedness,” while the latter is called “structural embeddedness” (27, 28), which we will refer to as “tie strength” and “embeddedness,” respectively, to avoid confusion.

Embeddedness is the notion that understanding the relationships or behaviors between individual actors requires an understanding of their social network structural configuration (17, 27). Embeddedness is theorized to be the social mechanism behind increased levels of trust, altruism, cooperation, and communications in relationships (29–33), with the underlying principle that within close-knit (i.e. highly embedded) networks, frequent social interactions between actors reinforce existing social norms, behaviors, and interactions (11, 30–32). Previous research shows that greater embeddedness encourages cooperative social behaviors such as favor exchanges (33, 34), relationship formation (35) (and even marriage (36)), dyadic trade (37), stable collaborations (38), and social conformance (26). Embeddedness also tends to be more stable than other properties of relationships since it enmeshes multiple actors and is less under the control by one pair of individuals, let alone a single individual (31). Consequently, one may also expect that embedded ties are more likely to be resilient sources of social support during emergencies.

In short, relationship strength is theorized to have two major aspects: the strength of the dyadic relationship (i.e. between two individuals), which is typically operationalized by tie strength and frequency of communications, and embeddedness, which is typically operationalized by the number of common friends shared by two individuals (i.e. the “overlap parameter,” OP) (19, 26, 31, 34). Prior research has often investigated the independent effects of tie strength versus embeddedness or even treated them as alternative measurements of relationship strength (19, 26). For example, both tie strength and embeddedness predict how much close individuals are and how much influence they exert on each other (26). However, there is less understanding of how tie strength and embeddedness can *interact* to jointly affect behavior.

Previewing our results briefly, we find that tie strength by itself is not the strongest predictor of relationship importance in postearthquake communications (for example, who an ego calls first, how quickly an ego makes the first call, and whether that person immediately reciprocates). Rather, the joint effect and interaction between tie strength and embeddedness best predicts emergency social network activation behavior; in other words, structural embeddedness moderates whether strong ties are important ties during the earthquake.

## Measures

Our analysis is based on individual-level mobile telecommunications data of 91,839 active local subscribers in the Ya’an prefecture of China (54,857 are in family plans, which have at least 2 members) during 2013 March 1 to May 31 (see Materials and methods for details and Table S1 for summary statistics). We operationalize tie strength using the frequency of communications between an ego and alter (19–21), i.e. voice call frequency, during the 4 weeks prior to the earthquake. We also use an alternative, rank-based measure of tie strength (21) in our supplementary analyses (Fig. S8, Table S4). We measured embeddedness using the number of common friends shared by two individuals (OP) (19, 26, 31, 34) for the same period.

Our empirical strategy is to link tie strength and embeddedness to dependent variables that are indicative of relationship strength or nature of relationship, which serve as benchmarks to compare the ecological validity of tie strength and structural embeddedness. In particular, we considered tie importance, communications latency, reciprocity, and family plan membership as dependent variables.

### Important ties

We define the first alter that an ego called after the earthquake as an “important tie.” We make no assumptions for motivation or purpose of communications, which may vary from informational to emotional support to coordination needs. For purposes of determining important ties, we only considered the first calls that were not to emergency and service hotlines. This definition is independent of network size (a network must contain more than one individual, so by definition everyone has at least one important tie).

### Network activation latency

**Fig. 1.**
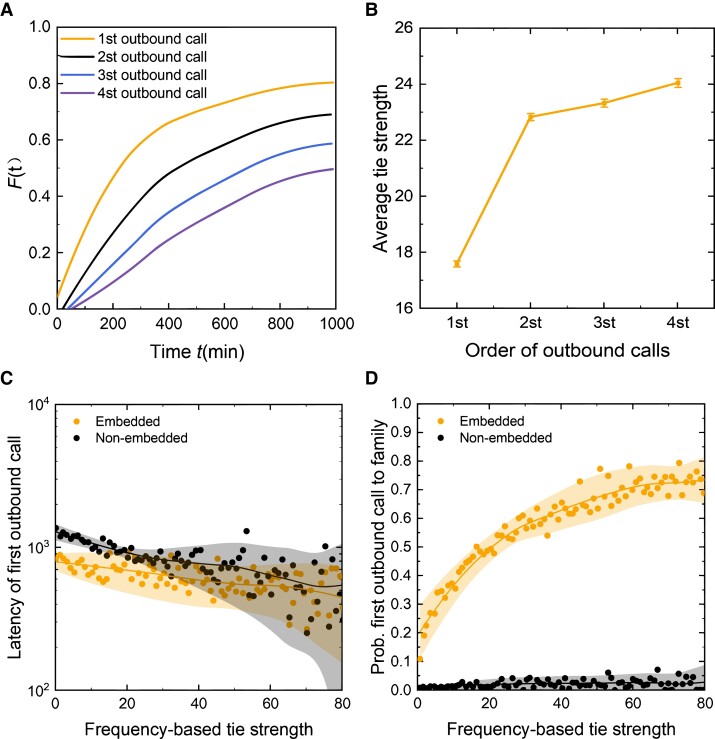
Tie importance, tie strength, and embeddedness. a) Temporal patterns of the first four outgoing calls after the earthquake. We interpret temporal order of calls as a revealed behavior that denotes relative importance of the social tie in the immediate aftermath of the earthquake. The first outgoing contact (i.e. important tie) is characterized by the greatest temporal urgency. b) Tie strength of the first four outgoing calls; the average tie strength of the important tie (17.6) is weaker than that of the second (22.8, *P* < 0.001), third, and fourth outgoing contacts. Overall, 40.65% of first outgoing calls were to weak ties (<3 communications in prior month), compared with 20.08% for the second outgoing contacts. In other words, tie strength does not necessarily predict rank order, and important ties can be weaker. c) The latency of social network activation is predicted together by both tie strength and embeddedness, and embedded ties tend to be activated more quickly than unembedded ties, especially for weaker ties. d) Likelihood that the first outgoing call is directed to family has a curvilinear relationship with embeddedness strength, which initially increases but declines after ∼7; the strongest predictor is simply the binary variable of whether family ties are embedded or unembedded. Embedded family ties are far more likely to receive first outgoing calls (*Pr* = 0.440 vs. 0.053, *P* < 0.001). Here, and in other figures, embedded ties are defined as ties that have a nonzero OP, i.e. share at least one common social tie.

We measured temporal latency until the ego's first outgoing call (i.e. hours since earthquake), which we refer to as activation latency. The intuition behind this measure is that the speed and urgency with which the ego contacts the important tie reflects how important it is for the ego to communicate with them and is some reflection of their closeness, intimacy, and relationship importance. The secondary implication of the measure is that it reflects the ego's speed and urgency in activating their social network as a whole. We also examined the latency of the second through fourth ranked contacts (Fig. 1a).

Overall, the temporal latency patterns comport with our expectations that communications immediately after a disaster should be characterized by urgency, which enhances our confidence in the data. The temporal distribution of first outgoing calls (i.e. important ties) is captured by an exponential distribution *p*(*x*) ∼ e*^ρx^*, with the scaling parameter *ρ* = −0.0061 (Fig. S5); in other words, the vast majority of first outgoing calls were made with low latency, soon after the earthquake. The temporal latency of earthquake victims’ first four outgoing calls (to the most important, second, third, and fourth most important ties) is characterized by a pattern of declining urgency for later communications, which can be seen in the steeper gradient of and greater spacing between earlier calls (Fig. 1a).

### Immediate reciprocity

Reciprocity is critical for the maintenance of cohesion, trust, relational stability, and social capital and is commonly used to operationalize social support and relational cohesiveness (24, 39, 40). Stronger social relationships, particularly those characterized by repeated interactions or in the context of favor exchange, are characterized by reciprocity of social actions (24, 33, 34). We used a strong measure of (immediate) reciprocity and define an important tie as reciprocal if the first call out and first call in were directed to and from, respectively, the same phone number (i.e. the important tie was also the first social tie to call the ego; this was the case for 13.28% of mobile subscribers in our data set). Such “immediate” reciprocity in communications, in advance of communications with other social ties, may be a strong signal of mutual concern and relationship depth.

### Family membership

Kinship networks may play outsized role during emergencies (41–44). One might wonder the extent to important ties is members of kinship rather than volitional (nonkinship) networks. We used membership in the telecom carrier's family plan as a proxy of whether or not the important tie was a family member. The measure is conservative because family members may have independent telecom plans; the measure is also relatively objective because the carrier requires national ID verification to qualify for family plan subscriptions. This dependent variable also allows us to test the degree to which family ties are strong or embedded.

It should be noted that the decision to first communicate with family or nonfamily members is relevant regardless of cohabitation. For example, for egos who live apart from family plan members, the calling decision may reflect that family member's relative importance to the ego. For families that live in the same home, intrafamily communications immediately after the earthquake may reflect the close-knit nature of the family unit and greater social coordination in postdisaster response (e.g. families may divide tasks, split up, and provide information updates).

## Results

### Exploratory analysis

We first investigate whether tie strength and embeddedness measures can predict tie importance (who an ego first calls after an earthquake). It is apparent that the most important tie is not necessarily the strongest tie (as measured by history or frequency of prior communications). Only 26.8% of important ties were also the strongest (most frequent) tie during predisaster time periods; 35.5% (19.8%) of important ties were ranked beyond top five (top ten) by tie strength. The discrepancy between tie importance and (rank of) tie strength, *r*, is characterized by a power law function, *p*(*r*) ∼ *r*^−*λ*^, with the scaling parameter *λ* = 1.089 (Fig. S5). Indeed, the average tie strength of the important tie (17.6) is weaker than that of the second (22.8, *P* < 0.001), third, and fourth outgoing contacts (Fig. 1b). In fact, 40.7% (20.1%) of important (second most important) ties had fewer than 3 communications with the ego in the prior month; in other words, many important ties were in fact “weak ties.”

**Fig. 2.**
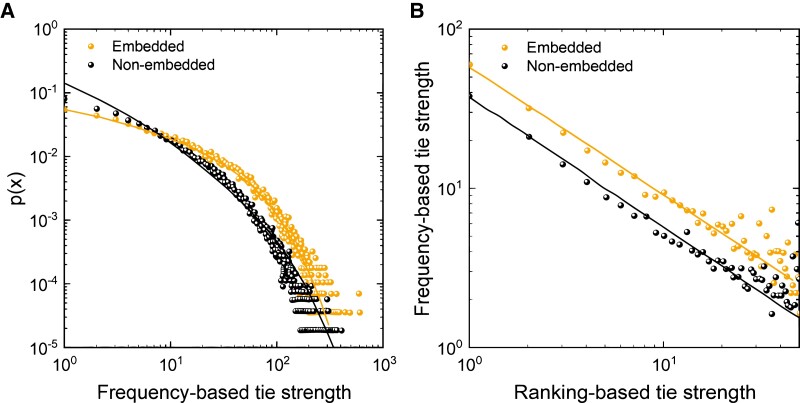
Relationship between important ties, tie strength, and embeddedness. a) Embedded important ties typically have stronger tie strength than unembedded important ties. The distributions for both are fitted by a power law function with an exponential cutoff, $p(x)=ax^{-b}e^{-cx}$, where *a* = 0.084, *b* = 0.515, and *c* = 0.018 for embedded ties and *a* = 0.342, *b* = 1.21, and *c* = 0.010 for unembedded ties. b) The relationship between ranking-based (*x*) and frequency-based (*y*) measures of tie strength both follow a power law, i.e. $y=ax^{-b}$, where *a* = 57.7 and *b* = 0.801 for embedded ties and *a* = 37.5 and *b* = 0.816 for unembedded ties; the two distributions are almost parallel (*b*-values are nearly equal), which suggests that embeddedness affects communications frequency for any given tie ranking (and vice versa) in a constant manner.

Embeddedness has a positive linear relationship with tie strength up to an OP of about 15, after which the relationship flattens (Fig. S6a). However, as a single independent variable, embeddedness is relatively more predictive of who an ego calls first after the earthquake. For example, egos have more embedded ties with the important tie (34.6%) than with the second (28.0%), third (26.5%), and fourth most important ties (25.21%), *P* < 0.001. When we dichotomously classify important ties into embedded and unembedded ties (Fig. 2a), the average tie strength of embedded ties is over two times stronger than that of unembedded ties (28.25 vs. 11.91, *P* < 0.001). For embedded ties, 35.27% of the strongest ties are important ties; for unembedded ties, only 22.14% of the strongest ties are important ties. Furthermore, at any level of tie strength, embedded important ties typically have a higher tie ranking than unembedded important ties (Fig. 2b). The relative tie strength of embedded and unembedded important ties both follow a power law distribution.

Quantitatively, greater embeddedness as measured by the OP is also predictive of likelihood that an important tie is a family plan member but only up to an upper bound of OP ∼7, after which it becomes a negative predictor (Fig. S6b). The simple dichotomous variable of whether the ego and the important tie are embedded (i.e. share at least one common friend) is also highly predictive of the characteristics of the important tie (Fig. 1c and d). Embeddedness is also indicative of how tight-knit families are; when embedded, egos in larger families are more likely to call family members first (Fig. S7a); when unembedded, family size has no effect on likelihood of intrafamily communications occurring first (Fig. S7b).

Tie strength is more predictive of communications behavior when assessed jointly with embeddedness. Structural embeddedness plays a significant moderating role between tie strength and activation latency; that is, strong ties are most important when they are also embedded. For example, at each level of tie strength (and tie ranking), and especially for weaker ties (i.e. <15), embedded ties have significantly lower latency (i.e. are called earlier) as compared with nonembedded ties (Fig. 1c).

The interaction is even more extreme for the family tie–dependent variable. Tie strength (and tie ranking) is a positive predictor of family tie activation only when the important tie is embedded and has no predictive power when the important tie is not embedded (Fig. 1d, Fig. S8b). We also observe a curvilinear relationship; the marginal effect of tie strength on likelihood of family tie activation is stronger for weak ties (i.e. <20) than strong ties (i.e. the curvature for the effect of embedded ties is steeper for weak ties than strong ties).

### Robustness check for inbound communications

We observe analogous patterns when predicting the source of the first incoming communications. First inbound communications are the initial manifestation of social support for the ego from their social network (Fig. S8). At each level of tie strength, embedded ties are relatively faster in calling the ego after the earthquake. Tie strength is not predictive of whether the first incoming call is from family unless the tie is embedded, in which case tie strength becomes a strong positive predictor.

### Hierarchical network of important ties

**Fig. 3.**
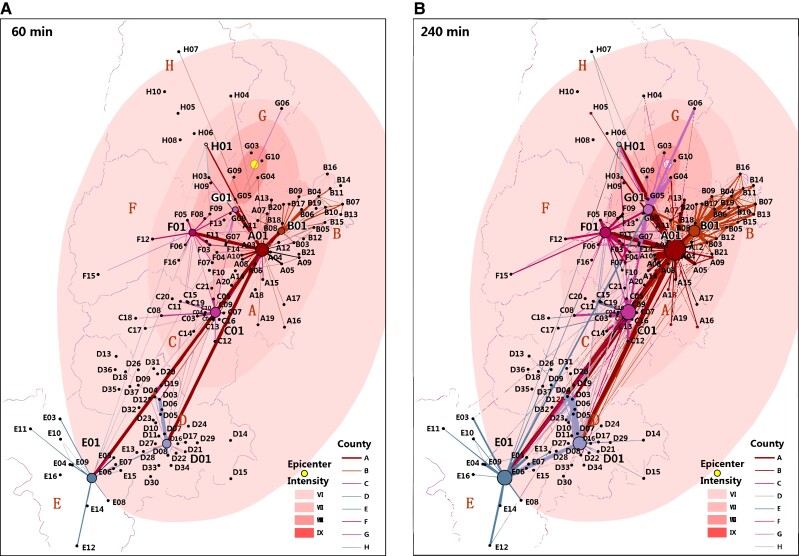
Geographic distribution of important ties. The social network backbone formed by the first outgoing calls in Ya’an occurring within (a) 60 min and (b) 240 min after the earthquake, transposed onto map of Ya’an. Edge width is proportional to the square root of the number of contacts between the two nodes; edge threshold set to 5. Node size is proportional to the square root of the number of self-referencing ties (i.e. bigger node denotes more ties directed to alters in the same node). Geographical coding (circles A to H) corresponds with Table S2. The 01 node under each geographical code represents the county seat (while the node A01 is the metropolitan city); other numbered nodes represent towns and villages in each county. The social network backbone with important ties corresponded with the geographic and administrative hierarchical structure of Ya’an prefecture. The prefecture-level city (A01) is clearly identified as the regional hub.

Our findings have macronetwork implications. When we consider the geographical distribution of destination of first outgoing calls (which defines the backbone of Ya’an's social network), we find that they form communities that map perfectly onto the social and geographical structure of the entire Ya’an prefecture (i.e. the macrostructure of society; Fig. 3, Figs. S3–S4). Notably, the giant connected network of important ties quickly links the stricken prefecture's towns and villages, which connect in a hierarchical manner, i.e. towns/villages are connected to the county seat but not with other towns/villages, especially in the initial time after the earthquake. We also applied a clustering identification method to important tie networks and confirmed the hierarchical structure of the prefecture community's social network backbone (Fig. S3). The role of strong versus weak ties in connecting communities is of theoretical importance, since weak ties are usually assumed to form the bridges connecting disparate communities in social networks (19). Here, we find that important ties, which are more analogous to strong ties, form the initial social network backbone that connects the disparate villages and towns to form the prefecture's macrosocial network in the immediate aftermath of disaster.

### Statistical models

The model-free analyses suggest that tie strength and embeddedness interact to drive postearthquake social network activation dynamics and that without embeddedness, tie strength is less predictive of relationship strength and depth. Next, we leverage the exogenous shock of the earthquake to formally test how tie strength and embeddedness affect communications dynamics after the earthquake. Specifically, we test how tie strength and embeddedness interact with the *level of earthquake intensity* to drive individuals’ postearthquake communications behavior. Our statistical models, besides statistically separating how tie strength versus embeddedness affects response to an exogenous shock, also address numerous potential sources of endogeneity (e.g. reverse causality and potential third variable confounders).

Our analyses exploit the fact that residents experienced different levels of earthquake intensity depending on their geographical location (Fig. S4). Earthquake intensity was random insofar that residents did not know nor could affect its intensity and location ex-ante. We divide residents into “severe” (earthquake magnitude VIII or above) and “mild” (magnitude VII or below) groups (as defined by Typical Maximum Modified Mercalli Intensity; Supplementary Material Section 2.1), and the main difference being whether there was physical damage to buildings or not. Presumably, experiencing physical damage (e.g. structural damage and economic loss), as opposed to only experiencing violent shaking without suffering damage, increased the need for social support, social coordination, and disaster response activities. Consequently, the interaction effect between the earthquake shock and tie strength versus embeddedness tests how greater (dyadic) tie strength and embeddedness, respectively, affects social dynamics in response to the earthquake.

Three separate sets of statistical models test the impact of tie strength, embeddedness, and their interaction terms with earthquake intensity on three different dependent variables:(i) activation latency (hours), (ii) whether the important tie reciprocates by calling back, and (iii) if the important tie is a family plan member or not. In each model, we operationalize tie strength using total frequency of communications between the ego and important tie in the 4 weeks prior to the earthquake and operationalize embeddedness using the number of mutual social ties between them (OP) in the same period. All models include individual fixed effects. Robustness checks include using a normalized measure of tie strength, roaming only samples, testing second through fourth calls, selecting for non-WeChat users, and using a nonparametric decision tree model (see Supplementary Material Section 4).

#### Model 1: activation latency

As presented in Table 1, the main effects of tie strength and embeddedness are both negative (*P* < 0.001). However, we observe a significant positive interaction effect between tie strength and embeddedness (*P* < 0.001), which suggests that ties that are both stronger and more embedded have slower activation latency. Earthquake intensity had no significant main effect; however, its significant positive two-way interactions with tie strength and embeddedness, and positive three-way interaction, show that earthquake intensity compounds the positive interaction between tie strength and embeddedness.

**Table 1.** Impact of tie strength and embeddedness on social network activation latency.

Dependent variable = latency of first outgoing call (hours)	Coef.	Robust SE	*z*	*P* > |*z*|	
Tie strength of important tie	−0.0020	<0.001	−246.11	<0.001	***
Embeddedness (OP) of important tie	−0.0453	<0.001	−512.71	<0.001	***
Earthquake intensity dummy (1 = severe)	0.0031	0.004	0.76	0.447	
Tie strength*embeddedness (OP) of important tie	0.0004	<0.001	459.05	<0.001	^***^
Tie strength*earthquake intensity dummy	0.0022	<0.001	172.00	<0.001	*
Embeddedness (OP) of important tie*earthquake intensity dummy	0.0023	<0.001	17.02	<0.001	***
Tie strength*embeddedness (OP) of important tie*earthquake intensity dummy	<0.0001	<0.001	12.54	<0.001	**
Important tie is family dummy	−0.1479	<0.001	−405.39	<0.001	*
Family plan size (1 to 5)	0.0060	<0.001	53.46	<0.001	
Degree centrality of ego	0.0003	<0.001	67.73	<0.001	*
Total call frequency of ego	−0.0037	<0.001	−3004.57	<0.001	***
Total text frequency of ego	0.0003	<0.001	308.97	<0.001	***
Internet usage frequency of ego	<0.0001	<0.001	20.73	<0.001	***
Total WeChat usage frequency of ego	−0.0002	<0.001	−92.35	<0.001	***
Total usage frequency of other instant messaging of ego	−0.0007	<0.001	−783.86	<0.001	***
Smartphone dummy (1 = smartphone user)	−0.1169	<0.001	−476.89	<0.001	***
Roaming dummy (1 = traveling outside of prefecture)	−0.5709	<0.001	−1320.25	<0.001	***
Rural dummy (1 = rural)	0.0328	<0.001	87.08	<0.001	***
Damage dummy (1 = cell towers damaged)	−0.1123	0.003	−33.95	<0.001	***
Constant	8.5800	0.003	3227.31	<0.001	***
Pseudo R-squared: 0.2174					
Number of obs = 89,907					

Poisson regression. Communications variables are average monthly data from 4 weeks before the earthquake. Fixed effects for 159 counties are included. ***P < .001, **P < .01, *P < .05.

**Fig. 4.**
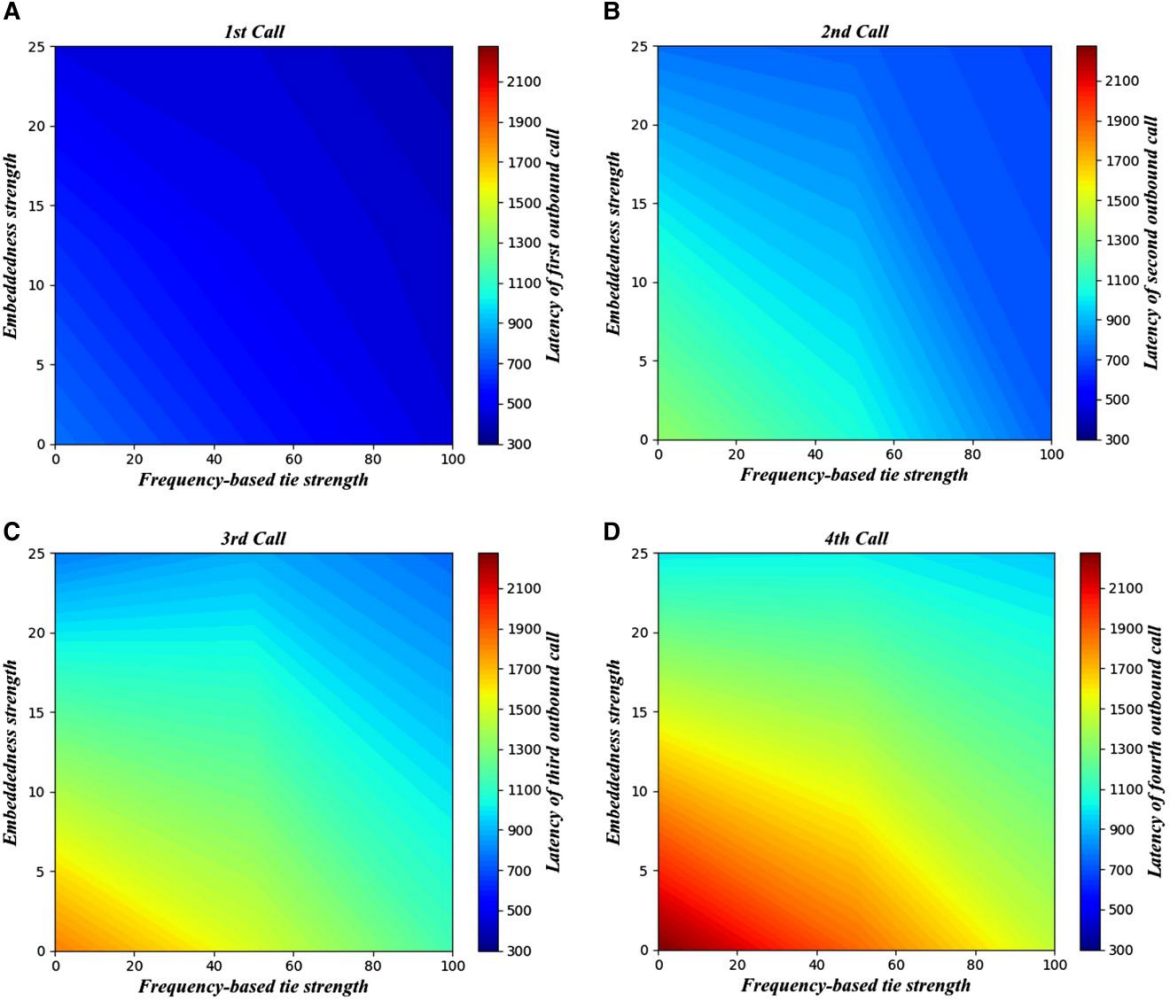
Predicting temporal latency of network activation. a–d) First to fourth outgoing calls, respectively. Greater tie strength and embeddedness both correspond to faster latency. The heat map represents the tradeoff between tie strength and embeddedness; one cannot fully substitute the other, i.e. important ties with high (low) tie strength and low (high) embeddedness; social ties suffer relatively greater latency than important ties with balanced tie strength and embeddedness. An interaction effect can be observed from the slight kink in the heat map, which denotes the “sweet-spot” for fastest latency (social ties with tie strength of ∼50 and embeddedness of ∼10–20).

Overall, we find that tie strength and embeddedness have separate as well as interactive effects on response latency. The results suggest diminishing returns (in response latency) for having both high tie strength and embeddedness. Although tie strength and embeddedness individually predict faster latency, ties that are both strong and embedded are slower to activate than ties that are high and low, or low and high, in tie strength and embeddedness, respectively. This implies that, for a given level of embeddedness, weaker ties will be activated relatively faster than stronger ties. This interaction effect is represented visually by the more significant difference between embedded and unembedded ties for weaker ties in Fig. 1c and also by the kinked lines in the Fig. 4 heat map.

#### Model 1 robustness checks

We observe the same significant positive three-way interaction for the temporal latency of the second, third, and fourth outgoing calls (Tables S10–S12). However, a notable difference for these calls is that embeddedness is a stronger predictor of temporal latency than tie strength. Furthermore, stronger tie strength actually predicts slower temporal latency for the third and fourth calls, particularly at higher earthquake intensity. This is consistent with the idea that important ties are often latent during normal times, when people may call the third or fourth ties more.

In addition, we use rank percentile as an alternative operationalization of tie strength and find consistent results (Table S4). We also tested the same models selecting only for customers who were roaming (i.e. out of town, *N* = 10,712; Table S5; the large number of roaming customers reflects that a significant number of family plan subscribers worked in other parts of the province or country). An alternative interpretation of Model 1 is that the most urgent first outgoing call is to core family members, and thus, the dependent variable might simply reflect if the ego lives with (or next door to) their core family members. However, this is unlikely to be the case since we observe the same significant positive two-way interactions with tie strength and embeddedness for roaming customers, who were all away from home. Overall, embeddedness is a better predictor for response latency than tie strength and weaker ties activated sooner for a given level of embeddedness.

#### Model 2: immediate reciprocity

We next examine how these factors affect the likelihood that the important tie is the same social tie who also made the first inbound call to the ego. Reciprocity serves as another behavioral marker of relationship importance. We find that tie strength and embeddedness have significant positive main effects on reciprocity (*P* < 0.001; Table 2). However, similar to Model 1, we also observe a significant negative three-way interaction between tie strength, embeddedness, and earthquake intensity (*P* = 0.023); once again, this negative term may reflect diminishing returns for tie strength and embeddedness. This implies that weaker ties will have more reciprocity than stronger ties for a given level of embeddedness.

**Table 2.** Impact of tie strength and embeddedness on reciprocity.

Dependent variable = *p*(reciprocal call)	Coef.	Robust SE	*z*	*P* > |*z*|	
Tie strength of important tie	0.1594	0.007	24.37	<0.001	***
Embeddedness (OP) of important tie	0.3206	0.025	12.73	<0.001	***
Earthquake intensity dummy (1 = severe)	−0.3117	0.329	−0.95	0.343	
Tie strength*embeddedness (OP) of important tie	−0.0198	0.010	−1.91	0.056	
Tie strength*earthquake intensity dummy	0.0139	0.011	1.31	0.189	
Embeddedness (OP) of important tie*earthquake intensity dummy	0.0318	0.039	0.82	0.414	
Tie strength*embeddedness (OP) of important tie*earthquake intensity dummy	−0.0369	0.016	−2.28	0.023	*
Important tie is family dummy	0.4749	0.016	29.01	<0.001	***
Family plan size (1 to 5)	−0.0559	0.006	−9.38	<0.001	***
Degree centrality of ego	−0.0033	<0.001	−8.61	<0.001	***
Total call frequency of ego	−0.0003	<0.001	−5.45	<0.001	***
Total text frequency of ego	−0.0001	<0.001	−1.26	0.208	
Internet usage frequency of ego	<0.0001	<0.001	−0.10	0.918	
Total WeChat usage frequency of ego	−0.0001	<0.001	−0.74	0.459	
Total usage frequency of other instant messaging of ego	<0.0001	<0.001	−0.60	0.547	
Smartphone dummy (1 = smartphone user)	−0.0186	0.012	−1.52	0.129	
Roaming dummy (1 = traveling outside of prefecture)	0.0747	0.017	4.46	<0.001	***
Rural dummy (1 = rural)	0.0210	0.020	1.05	0.294	
Damage dummy (1 = cell towers damaged)	−0.1748	0.282	−0.62	0.535	
Constant	−0.9365	0.206	−4.54	<0.001	***
Pseudo R-squared: 0.0915					
Number of obs = 91,839					

Probit model. Communications variables are average monthly data from 4 weeks before the earthquake. Fixed effects for 159 counties are included. To address the potential quasi-complete separation problem between the dependent variable and these two key independent variables, we categorize tie strength into five ordered intervals which contain similar number of data points, which are 0, (1, 10), (11, 20), (21, 30), and 31 or above, and categorize embeddedness as 0 or 1 (when embeddedness is larger than 0).

***P < .001, **P < .01, *P < .05.

#### Model 3: activation of family ties

Next, we use a probit model to predict whether an ego's first call is to family plan members or not. For this analysis, we only select egos who are in family plans and have at least another family plan member in our data set (*N* = 54,857; Table 3). The decision to call family plan members first was not a default; in fact, initial communications were dominated by calls to nonfamily plan members. Of the users who were not roaming, only 16.21% of first outgoing calls were to other family plan members; users who were roaming, and thus away from home, were 50% more likely to call other family plan members (24.35%).

**Table 3.** Predicting if the first outgoing call after earthquake is to family.

Dependent variable = *p*(important tie is family plan member)	Coef.	Robust SE	*z*	*P* > |*z*|	
Tie strength of important tie	0.0143	<0.001	40.39	<0.001	***
Embeddedness (OP) of important tie	0.1497	0.003	44.33	<0.001	***
Earthquake intensity dummy (1 = severe)	−0.7847	0.496	−1.58	0.114	
Tie strength*embeddedness of important tie	−0.0007	<0.001	−17.05	<0.001	***
Tie strength*earthquake intensity dummy	0.0025	0.001	4.60	<0.001	***
Embeddedness (OP) of important tie*earthquake intensity dummy	−0.0420	0.005	−8.56	<0.001	***
Tie strength*embeddedness (OP) of important tie*earthquake intensity dummy	0.0002	<0.001	3.03	0.002	**
Family plan size (1 to 5)	0.2168	0.009	25.14	<0.001	***
Degree centrality of ego	<0.0001	<0.001	−0.04	0.969	
Total call frequency of ego	−0.0014	<0.001	−23.34	<0.001	***
Smartphone dummy (1 = smartphone user)	−0.0002	<0.001	−4.41	<0.001	***
Internet usage frequency of ego	<0.0001	<0.001	−1.14	0.253	
Total WeChat usage frequency of ego	<0.0001	<0.001	−0.08	0.937	
Total usage frequency of other instant messaging of ego	<0.0001	<0.001	−0.58	0.564	
Smartphone dummy (1 = smartphone user)	−0.0538	0.014	−3.85	<0.001	***
Roaming dummy (1 = traveling outside of prefecture)	0.3021	0.019	16.25	<0.001	***
Rural dummy (1 = rural)	0.0381	0.024	1.59	0.111	
Damage dummy (1 = cell towers damaged)	0.5442	0.429	1.27	0.205	
Constant	−1.6370	0.307	−5.34	<0.001	***
Pseudo R-squared: 0.2148					
Number of obs = 54,857					

Probit model. Communications variables are average monthly data from 4 weeks before the earthquake. Fixed effects for 159 counties are included. ***P < .001, **P < .01, *P < .05.

Consistent with previous models, tie strength and embeddedness are both positive predictors of whether the important tie is a family plan member (*P <* 0.001). However, the overall set of interaction effects is more complex: tie strength and embeddedness have a significant negative interaction effect (*P <* 0.001); earthquake intensity and tie strength have a positive interaction effect (*P* = 0.001); earthquake intensity and embeddedness have a negative interaction effect (*P* = 0.005); the three-way interaction effect is positive (*P <* 0.001). Overall, the negative interaction effect between tie strength and embeddedness is more significant than other interaction terms and is the dominating effect. As Fig. 1d illustrates, for the embedded ties, tie strength has a diminishing marginal effect on the likelihood of calling a family member. That is, the marginal effect of tie strength in predicting activation of family ties is greater for relatively weaker ties (than stronger ties).

### Roaming and internet robustness check

We observe similar results when only selecting for roaming customers (who are also family plan members, *N* = 10,794; Table S16); it is thus unlikely that our model only predicts likelihood of family cohabitation. We also observe the same pattern of results when selecting only for customers who subscribed to internet services and used mobile apps (Table S15), which suggests that our results are unlikely to be driven by technological sophistication or internet access–related factors (e.g. if only the elderly or less technologically adept drove the original effects).

### WeChat robustness check

We explore the impact of WeChat adoption on our data in Supplementary Material Section 1; we found that WeChat did not affect voice call usage, the focal behavior in our analyses, during our study period. We also conduct an additional empirical analysis by excluding those who previously used WeChat before the earthquake in our mobile phone data (the carrier observes app data usage for billing purposes). This additional analysis yields consistent results for activation latency, immediate reciprocity, and family plan tie activation prediction (Tables S17–S19).

### Replication using decision trees

As an alternative analysis, we use a decision tree model, a nonparametric supervised learning method for prediction, to explore the predictive capacity of tie strength and embeddedness. Decision trees can help discover interactions among independent variables, in which case variables would appear together in a traversal path. The results illustrate that tie strength and embeddedness interact to predict the emergency communications behavior and yield consistent results as our regression models (Table S20, Figs. S12–S14).

Finally, to provide greater interpretability for our results, we use a random forest model to generate importance scores for tie strength and embeddedness for Model 3 (see Supplementary Material Section 4.7). The scores for variable importance highlight that tie strength and embeddedness are the two most significant predictors, with importance scores of 57.32 and 29.62, respectively.

## Discussion

During disasters, social ties can function as social capital and serve as a means for victims to access to information, resources, and emotional support (45, 46). Overall, our findings reveal the subtle behavioral and relational aspects of social network activation in the extreme context of an earthquake. Our main conceptual contribution is in investigating the relationship between two of the most widely used social network metrics, tie strength and embeddedness, and their joint effects on real social behavior during the disaster. The most common theoretical conceptualization of relationship strength defines strong ties as emotionally close relationships characterized by frequent interactions; this perspective and common empirical operationalization, based on interaction intensity, is the most typical measurement of social relationship capital at the dyadic level (17–22, 27) and underpins theories of interpersonal relationships (19–21), human cooperation (23, 24), labor economics (17, 18, 27), information diffusion (25), and social influence (26). Accordingly, one might expect disaster victims to also first communicate with their strongest ties. However, we find that the social ties with the highest tie strength during normal times often were not the most important tie during the disaster. We show that whether a social tie is prioritized during emergency also depends on the interaction between a relationship's dyadic intensity and its structural embeddedness with other relationships in the ego network.

Whereas previous research has considered tie strength and embeddedness to be alternative measures of relationship strength (19, 26), there is less understanding in how they interact to jointly predict social behavior or relationship importance. We find that although tie strength and embeddedness are highly correlated, both with each other and also with tie importance (and reciprocity and family membership), they do not fully overlap. The differing interaction effects across our models (and for different dependent variables) show that their relationship is complex and context dependent. This point is also illustrated by differences in the decision tree models (Figs. S12–S14); although tie strength and embeddedness appear together in the traversal paths, the parent nodes differ across different contexts.

A unifying theme across all of our model results is that tie strength and embeddedness have a moderating relationship on one another. In the case of predicting whether the important tie is a family member, Fig. 1d visually illustrates (and simplifies) the significant interaction effect of Table 3. Here, we find that the embeddedness of important ties is a necessary condition for tie strength to have any predictive power at all. In other words, tie strength has no predictive power if it is not embedded. In another less extreme case, Fig. 1c shows that although tie strength predicts temporal latency, the distribution of the relationship differs for embedded versus nonembedded ties. Overall, the interaction effect of embeddedness and tie strength has an opposite direction to their main effects, which implies that (un-)embeddedness has a constraining effect on tie strength and also that weak ties can be more important when embedded.

The moderating effect of embeddedness on tie strength has implications for interpreting the theoretical relationship between relational and structural embeddedness. In providing an initial definition of tie strength and structural embeddedness, Granovetter originally remarked that “the degree of overlap of two individuals’ friendship networks varies directly with the strength of their tie to one another” (7). This basic idea also explains why during the social process of network formation, people with strong ties are more likely to have more common friends [from sharing more foci of activities with one another (30)] and vice versa. Our results bring empirical findings, which had hitherto largely separated tie strength and embeddedness, full circle to show that dyadic ties need to be embedded within other shared relationships in order for tie strength to be meaningful (at least during emergencies).

Central to our analyses is the use of a quasi-experimental context, where behavioral patterns are themselves meaningful, to create behaviorally inferred measures of relationship strength, for example, rank choice of social preference (i.e. tie importance), temporal urgency, and social reciprocity. Our research approach highlights the value of using nonregular events—such as natural disasters—to unveil features and relationships in social networks that are latent during normal times. We make a methodological contribution by demonstrating the potential for using behavior during such contexts (when victims may mobilize their social and relationship capital) to measure and benchmark constructs such as relationship strength. Although we recognize that using measures such as communications frequency to infer relationship strength is a matter of practicality, we hope to illuminate the limitations of relying purely on historical behavioral frequency to predict the state of existent relationships (or future behaviors), without sufficiently considering social context. Future research can consider other special situations (for example, holidays or job loss) that may reveal relationship ground truths and the mechanistic complexities underlying social relationships. Future research can also explore the differences between behavioral and frequency measures; for example, we find that the statistical properties of revealed social tie importance do not fully correspond to frequency-based measures of tie strength, which have exponential decay rather than linear relationships with each other.

Given that social network data is often temporal (e.g. with time stamps) and spatial (e.g. cell tower location), future research may also investigate the temporal–spatial characteristics of emergency communications for the entire macronetwork to understand how collective behavior evolves in disaster contexts. Such analyses, if linked to recovery outcomes, may reveal how individual-level relationship capital aggregates across space and time to provide macrolevel social capital (22), which can abet society's recovery from negative shocks.

Our also research provides insights on the social network foundations of social resilience to disaster. Although weak ties have been proposed to provide structural interconnectivity (and resilience) in networks by embedding disparate communities (17–19, 27), our results show that the backbone network of important ties is sufficient to span the disparate towns and villages of the stricken prefecture. Conceptually, this raises questions as to whether there is necessarily always a tradeoff between relationship strength and reach across a network (e.g. the relationship between relationship strength and information diffusion). Part of the puzzle may lie in the motivation of different types of ties to communicate, seek information, and provide support during an emergency context (as opposed to diffusing information about job prospects). Our results also suggest that there may be a difference between “normal” social capital (that is deployed in normal, nonemergency contexts) and the resilient, emotionally strong social capital that is tapped during emergencies. Understanding the conceptual and actual basis of social capital in response to disasters has important implications for public policy as communities that have more social capital (built from community members’ social ties) typically recover faster after disasters, a finding observed in both the developed and developing worlds (4, 5, 9).

## Materials and methods

### Data

We used 3 months of anonymized individual-level telecommunications records (2013 March 1 to May 31) of 91,839 active subscribers of a major Chinese mobile telecommunications carrier, who were registered locally in the Ya’an region of Sichuan (see Table S2 for geographical information). A total of 95.7% of subscribers in the data set subscribed to a broadband or landline telephone service; associated addresses were thus unlikely to have been fraudulent. The data included time-stamped records of individuals’ voice calls, text messages (SMS), mobile internet usage, mobility (tower access), demographics, and customer data (e.g. phone model, spending, and family plan membership) (see Table S1 for summary statistics). Overall, 54,857 users in the data set were in family plans (19,305 are in 2-person plans; 28,038 are in 3-person plans). The earthquake occurred at 08:02 AM on 2013 April 20. Most preearthquake social network variables (e.g. tie strength or embeddedness during normal times) were calculated from March 2013 data. See Supplementary Material Section 1 for checks on impact of WeChat adoption, which did not affect voice call usage and the validity of our operationalizations (based on voice call data).

### Statistical models

#### Model 1: effect of tie strength and embeddedness on activation latency of social network

Model 1 explores how tie strength and embeddedness affects latency of social network activation. Since the dependent variable is a count variable (hours), we use a Poisson regression.

Model 1.1 is the baseline model:


$$E(y_{i}|X_{i})=\exp(\alpha_{1}\mathrm{Ti}e_{i}+\alpha_{2}\mathrm{Embeddednes}s_{i}+\alpha_{3}\mathrm{Ti}e_{i}\cdot \mathrm{Embeddednes}s_{i}+C_{i}\beta ),$$

where $y_{i}$ is latency (hours) for individual *i*; $X_{i}$ is a vector of independent variables including our key explanatory variables, $\mathrm{Ti}e_{i}$ and $\mathrm{Embeddednes}s_{i}$; and $C_{i}$ is a vector of control variables. $\mathrm{Ti}e_{i}$ and $\mathrm{Embeddednes}s_{i}$ are the tie strength and embeddedness, respectively, between individual *i* and the activated social tie. $C_{i}$ includes family plan member dummy, the ego's family size (number of family plan members), degree centrality, previous call frequency, text frequency, internet usage frequency, WeChat usage frequency, instant messaging (other than WeChat) usage frequency, smartphone use dummy, roaming (out of town) during earthquake dummy, rural address dummy, local cell tower damage dummy, and 159 county fixed effects.

The same model specifications are used in various robustness checks including when we use rank percentile as an alternative measurement for tie strength, only include roaming users, or predict temporal latency of second, third, and fourth calls (Tables S3–S12).

Model 1.2 is similar to Model 1.1 but also includes two-way interaction terms between the “severe” earthquake group dummy with tie strength and embeddedness, as well as a three-way interaction term:


$$\begin{array}{rl} E(y_{i}|X_{i}) & =\exp(\alpha_{1}\mathrm{Ti}e_{i}+\alpha_{2}\mathrm{Embeddednes}s_{i}+\alpha_{3}\mathrm{Sever}e_{i}\\ & \;+\alpha_{4}\mathrm{Ti}e_{i}\cdot \mathrm{Embeddednes}s_{i}\\ & \;+\alpha_{5}\mathrm{Ti}e_{i}\cdot \mathrm{Sever}e_{i}+\alpha_{6}\mathrm{Embeddednes}s_{i}\cdot \mathrm{Sever}e_{i}\\ & \;+\alpha_{7}\mathrm{Ti}e_{i}\cdot \mathrm{Embeddednes}s_{i}\cdot \mathrm{Sever}e_{i}+C_{i}\beta ), \end{array}$$

where $\mathrm{Ti}e_{i}$, $\mathrm{Embeddednes}s_{i}$, and $C_{i}$ are the same variables as in Model 1.1. $\mathrm{Sever}e_{i}$ is a dummy variable indicating whether individual *i* belongs to the “severe” earthquake group (1 = yes). $\alpha_{7}$ is the key parameter of interest which measures whether the interaction effect between tie strength and embeddedness (on social network activation latency) significantly varies across the two different earthquake intensity groups. We use the same model specifications to predict latency of second, third, and fourth calls (Tables S6–S12).

#### Model 2: effect of tie strength and embeddedness on social reciprocity

Model 2 examines how tie strength and embeddedness affect social reciprocity, i.e. whether an ego receives a call back. We use a probit model to account for the dichotomous dependent variable. Model 2.1 is the baseline model:


$$\begin{array}{rl} \mathrm{Pr}(\mathrm{Reciprocit}y_{i}=1|X_{i}) & =\Phi (\alpha_{1}\mathrm{Ti}e_{i}+\alpha_{2}\mathrm{Embeddednes}s_{i}+\alpha_{3}\mathrm{Sever}e_{i}\\ & \;+\alpha_{4}\mathrm{Ti}e_{i}\cdot \mathrm{Embeddednes}s_{i}\cdot \mathrm{Sever}e_{i}+C_{i}\beta ). \end{array}$$

Model 2.2 also includes the earthquake intensity interaction terms:


$$\begin{array}{rl} \mathrm{Pr}(\mathrm{Reciprocit}y_{i}=1|X_{i}) & =\Phi (\alpha_{1}\mathrm{Ti}e_{i}+\alpha_{2}\mathrm{Embeddednes}s_{i}+\alpha_{3}\mathrm{Sever}e_{i}\\ & \;+\alpha_{4}\mathrm{Ti}e_{i}\cdot \mathrm{Embeddednes}s_{i}+\alpha_{5}\mathrm{Ti}e_{i}\cdot \mathrm{Sever}e_{i}\\ & \;+\alpha_{6}\mathrm{Embeddednes}s_{i}\cdot \mathrm{Sever}e_{i}\\ & \;+\alpha_{7}\mathrm{Ti}e_{i}\cdot \mathrm{Embeddednes}s_{i}\cdot \mathrm{Sever}e_{i}+C_{i}\beta ), \end{array}$$

where $\mathrm{Reciprocit}y_{i}$ is a dummy variable indicating whether individual *i* receives subsequent call from the social tie they called (1 = yes). $X_{i}$, $\mathrm{Ti}e_{i}$, $\mathrm{Embeddednes}s_{i}$, and $C_{i}$ are the same variables as defined in Model 1. Φ is the cumulative density function of the standard normal distribution.

#### Model 3: effect of tie strength and embeddedness on family tie activation

We use a probit model to predict probability an ego first calls a family plan member or not after the earthquake. Model 3.1 is the baseline model:


$$\begin{array}{rl} \mathrm{Pr}(\mathrm{Famil}y_{i}=1|X_{i}) & =\Phi (\alpha_{1}\mathrm{Ti}e_{i}+\alpha_{2}\mathrm{Embeddednes}s_{i}+\alpha_{3}\mathrm{Sever}e_{i}\\ & \;+\alpha_{4}\mathrm{Ti}e_{i}\cdot \mathrm{Embeddednes}s_{i}\cdot \mathrm{Sever}e_{i}+C_{i}\beta ). \end{array}$$

Model 3.2 includes the earthquake intensity interaction terms:


$$\begin{array}{rl} \mathrm{Pr}(\mathrm{Famil}y_{i}=1|X_{i})= & \Phi (\alpha_{1}\mathrm{Ti}e_{i}+\alpha_{2}\mathrm{Embeddednes}s_{i}+\alpha_{3}\mathrm{Sever}e_{i}\\ & +\alpha_{4}\mathrm{Ti}e_{i}\cdot \mathrm{Embeddednes}s_{i}+\alpha_{5}\mathrm{Ti}e_{i}\cdot \mathrm{Sever}e_{i}\\ & +\alpha_{6}\mathrm{Embeddednes}s_{i}\cdot \mathrm{Sever}e_{i}\\ & +\alpha_{7}\mathrm{Ti}e_{i}\cdot \mathrm{Embeddednes}s_{i}\cdot \mathrm{Sever}e_{i}\\ & +C_{i}\beta ), \end{array}$$

where $\mathrm{Famil}y_{i}$ is a dummy variable indicating whether individual *i* activates family social support (1 = yes) in the aftermath of earthquake. $X_{i}$, $\mathrm{Ti}e_{i}$, $\mathrm{Embeddednes}s_{i}$, and $C_{i}$ are the same variables as defined in Model 1. Φ is the cumulative distribution function of the standard normal distribution.

## Supplementary Material

pgad358_Supplementary_Data

## Data Availability

Our contract with the telecom carrier prevents us from sharing the full telecommunications data set publicly. Sample data and aggregated statistics for replication and academic research purposes are available from the corresponding author (jmjia@cuhk.edu.cn) on reasonable request.
