# Supplementary material for: Emergency communications after earthquake reveal social network backbone of important ties
Source: PNAS Nexus. 2023 Nov 2;2(11):pgad358. doi: 10.1093/pnasnexus/pgad358 (PMC10658761; doi:10.1093/pnasnexus/pgad358)
Supplement: pgad358_Supplementary_Data [file pgad358_supplementary_data.pdf]

Supplementary Information for

Emergency Communications after Earthquake Reveal Social Network Backbone of Important Ties

Jayson S. Jia[1*], Yiwei Li[2], Sheng Liu[2], Nicholas A. Christakis[3], Jianmin Jia[4,5*]

[1] Faculty of Business and Economics, The University of Hong Kong, Hong Kong SAR, China.

[2] Department of Marketing & International Business, Faculty of Business, Lingnan University, Hong Kong SAR, China.

[3] Yale Institute for Network Science, Yale University, New Haven, CT, U.S.A.

[4] Shenzhen Finance Institute, School of Management and Economics, The Chinese University of Hong Kong, Shenzhen, China.

[5] Shenzhen Institute of Artificial Intelligence and Robotics for Society, Shenzhen, China

[*]Corresponding authors. Email: jmjia@cuhk.edu.cn (JMJ); jjia@hku.hk (JSJ)

This PDF file includes:

- Supplementary text
- Figures S1 to S14
- Tables S1 to S20
- SI References

Table of Contents

1 Mobile Telecom Data 3
2 Earthquake Background and Geography 6
2.1 Earthquake Background 6
2.2 Geographic location of important ties 6
2.3 Local versus non-local communications 9
3 Supporting Figures 10
3.1 Supporting figures for figures in Main Text 10
4.2 Incoming vs. outgoing communications 14
4 Supporting Tables and Models 16
4.1 Baseline models for response latency. 16
4.2 Three-way-interaction models including earthquake intensity 16
4.3 Reciprocity models 16
4.4 Predicting if important tie is a family plan member 16
4.5 Robustness checks using non-WeChat users 17
4.6 Decision tree analysis 17
4.7 Random forest model 18
SI References 40

1 Mobile Telecom Data

We used data centered on the 2013 Ya'an earthquake (Ms 7.0) in Sichuan, China as a situational test of what factors predicted and drove post-disaster communications. A basic assumption for our research, and existing literature, is that mobile phone data (and voice call data) can reflect the structure and characteristics of real world social relationships and networks. Indeed, an extensive literature has shown that mobile phone data, and call detail records (CDR), can reflect the strength of interpersonal social relationships (e.g., Barabási 2005, Onnela et al. 2007, Eagle, Pentland, Lazer 2009, Jo et al. 2014, Palchykov et al. 2012, Saramäkia et al. 2014). CDR includes details of telephone calls and other telecommunications exchanges (e.g., SMS, internet usage, roaming status) used for billing purposes. Table S1 provides the basic summary statistics of the dataset used for this study. The carrier also observes data usage for individual apps (frequency, MB). Our analysis focuses on voice call data (e.g., source number, destination number, time, duration of call, etc.), which serve as the DV.

Table S1. Statistics summary.

Variable name	N	Mean	Median	SD	Min	Max
Latency of first outgoing call (minutes)	91,839	967.34	215	2,108.02	0	15,325
Reciprocal call	91,839	0.13	0	0.34	0	1
Important tie is family plan member	91,839	0.17	0	0.38	0	1
Tie strength of important tie	91,839	15.56	4.00	29.15	0	592
Embeddedness (OP) of important tie	91,839	1.42	0	3.28	0	94
Earthquake intensity dummy (1 = severe)	91,839	0.41	0	0.49	0	1
Family plan size (1 to 5)	91,839	2.08	2.00	1.06	1	5
Degree centrality of ego	91,839	34.29	23.0	40.38	0	2,852
Total call frequency of ego	91,839	208.80	141	225.74	0	4,898
Total text frequency of ego	91,839	49.97	7.00	139.48	0	4,689
Internet usage frequency of ego	91,839	122.12	11.0	357.93	0	26,923
Total WeChat usage frequency of ego	91,839	19.98	0	71.52	0	1,806
Total usage frequency of other instant messaging of ego	91,839	83.03	0	231.86	0	4,014
Smartphone dummy (1 = smartphone user)	91,839	0.49	0	0.50	0	1
Roaming dummy (1 = traveling outside of prefecture)	91,839	0.12	0	0.32	0	1
Rural dummy (1 = rural)	91,839	0.43	0	0.49	0	1
Damage dummy (1 = cell towers damaged)	91,839	0.03	0	0.18	0	1

WeChat Data

Given the ubiquity of WeChat as a communications platform in China in recent years, one might wonder if the rapid adoption of WeChat across China affected the external validity of the CDR data, i.e., whether WeChat led to a substantial substitution effect for telecom usage (and voice calls) during the time of our study (March to June 2013). Our data suggests this was not the case; our models controlled for WeChat usage, and we later separately ran a robustness check using customers who did not use WeChat (Tables S20-S22). Here, we provide some intuitive explanation for why WeChat's growth in China had little effect on our study results. Firstly, WeChat 'only' had 194 million of monthly active users (MAU) by the end of Quarter 1 of 2013, who were mostly in major cities.

Secondly, using aggregate telecom usage data (2014-2017) for the Ya'an region provided by the carrier, we find evidence that WeChat substituted outgoing SMS messages but not voice calls. Indeed, WeChat did not introduce its VOIP (voice call) service until 2016 (i.e., a direct substitute for voice calls), several years after our study period.

Figures S1 to S2 illustrate how voice call and SMS usage changed between January 2014 and July 2017. During this period, the number of WeChat users grew dramatically and reached 963 million MAU by Q3 2017. If WeChat functioned as a direct substitute for voice calls, then we should have observed a correspondingly dramatic decline in voice call usage between January 2014 and July 2017. However, call duration, for both in- and out-bound calls was very stable over time; call frequency saw a slightly increase over this time period (Fig S1). However, we observed a declining trend in the frequency of outgoing text messages (Fig S2), which suggests WeChat may have substituted for text message communications (but not voice calls). We observed a slight increase in in-bound text messages, which was likely driven by increases in business SMS solicitations, which were commonplace at the time.

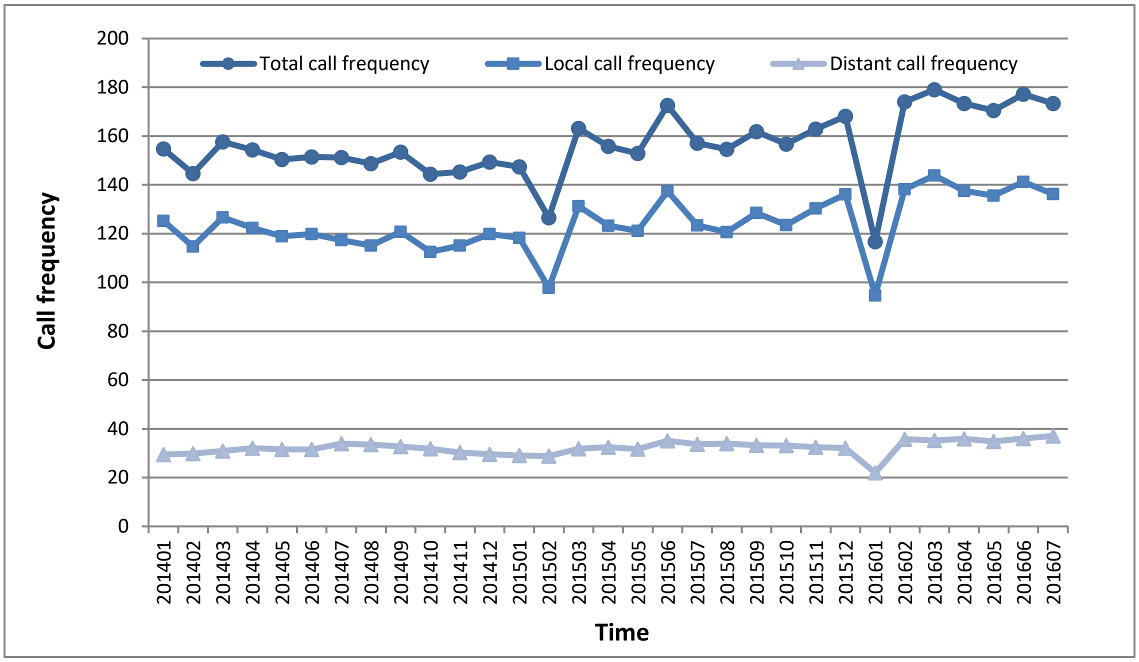

Fig. S1. Stability of voice call usage over time. Voice call usage frequency remains stable and exhibits growth over time for both local and long-distance calls. The seasonal dips in communications frequency correspond to the Lunar New Year holidays, when almost everyone is off work and visiting family.

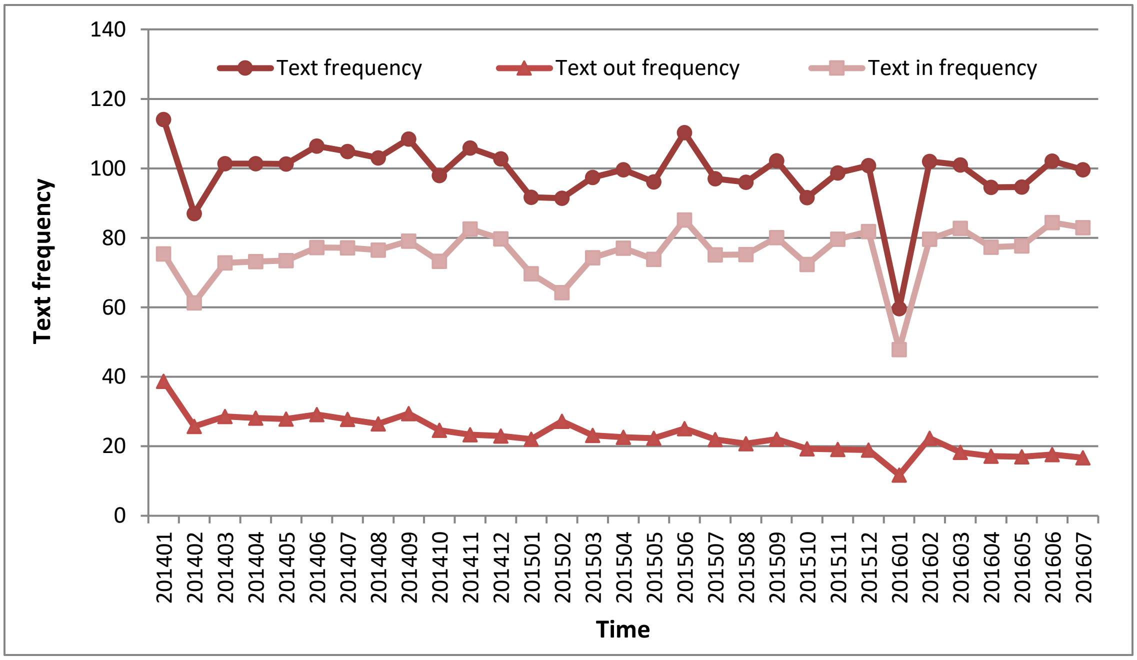

Fig. S2. Declining trend of text message (SMS) usage over time. In contrast to voice call usage, we observe a declining trend for outbound SMS usage over time; this trend largely occurred after our study period.

2 Earthquake Background and Geography

2.1 Earthquake Background

The Ya'an earthquake occurred at 08:02 (Beijing Time, UTC +8:00) on April 20 (Saturday), 2013, with the epicenter located in Lushan County, Ya'an, Sichuan, about 116 km from Chengdu, Sichuan (in southwest China). The China Earthquake Data Center placed the magnitude of the earthquake at M_s 7.0[1].

We used Earthquake intensity metric (Typical Maximum Modified Mercalli Intensity), a categorical measure reflecting physical damage sustained, to measure the earthquake's impact (US Geological Survey). Note that intensity differs from Richter scale or peak ground acceleration which measure amount of energy released. 4.0-4.9 on the Richter scale roughly corresponds to IV-V intensity, 5.0-5.9 to VI-VII intensity, 6.0-6.9 to VII-IX intensity, 7.0 and higher to VIII or higher. The intensity of the Ya'an earthquake ranged from V to IX. Our analyses separate the effect of the earthquake into two levels: Magnitude VI and below, and magnitude VII and above, with the logic that everyone in the dataset experienced a major earthquake, and that the primary difference in impact was whether there was physical damage or not (i.e., magnitude VII and above).

Table S2. Demographics of the Ya'an region.

City/County	Yucheng	Mingshan	Yingjing	Hanyuan	Shimian	Tianquan	Lushan	Baoxing	Total
County Code	A	B	C	D	E	F	G	H	8
Urban:	13,744	6,493	6,264	8,305	6,718	4,556	3,057	3,265	52,402
Rural:	5,986	10,974	3,180	8,256	2,153	3,360	4,086	1,442	39,437
Population	347,000	276,000	151,000	328,000	123,000	154,000	120,000	59,000	1,558,000
Relative user population* (%)	5.69%	6.33%	6.25%	5.05%	7.21%	5.14%	5.95%	7.98%	5.89%

*number of users in county / population of county

2.2 Geographic location of important ties

Ya'an prefecture has 8 counties and each county has many towns/villages. Table S2 provides population and the relative population of users for each county. We mapped the spatial distribution of the first outgoing calls (i.e., important ties) in order to explore the geographic properties of social network activation under risk (Fig. 3 in main text). We applied an algorithm (Ball et al. 2011) that detected how sub-communities of the network were clustered. The algorithm uses a principled statistical approach using generative network models, which divide edges into different groups (clusters) that maximize a

[1] https://en.wikipedia.org/wiki/2013_Lushan_earthquake

valuation function. The size of node is proportional to the square root of the number of activated users in the same node (i.e., self-referencing ties) at that time point (bigger node denotes relatively more important ties within same locale). Edge width is also proportional to the square root of the number of links between the two nodes.

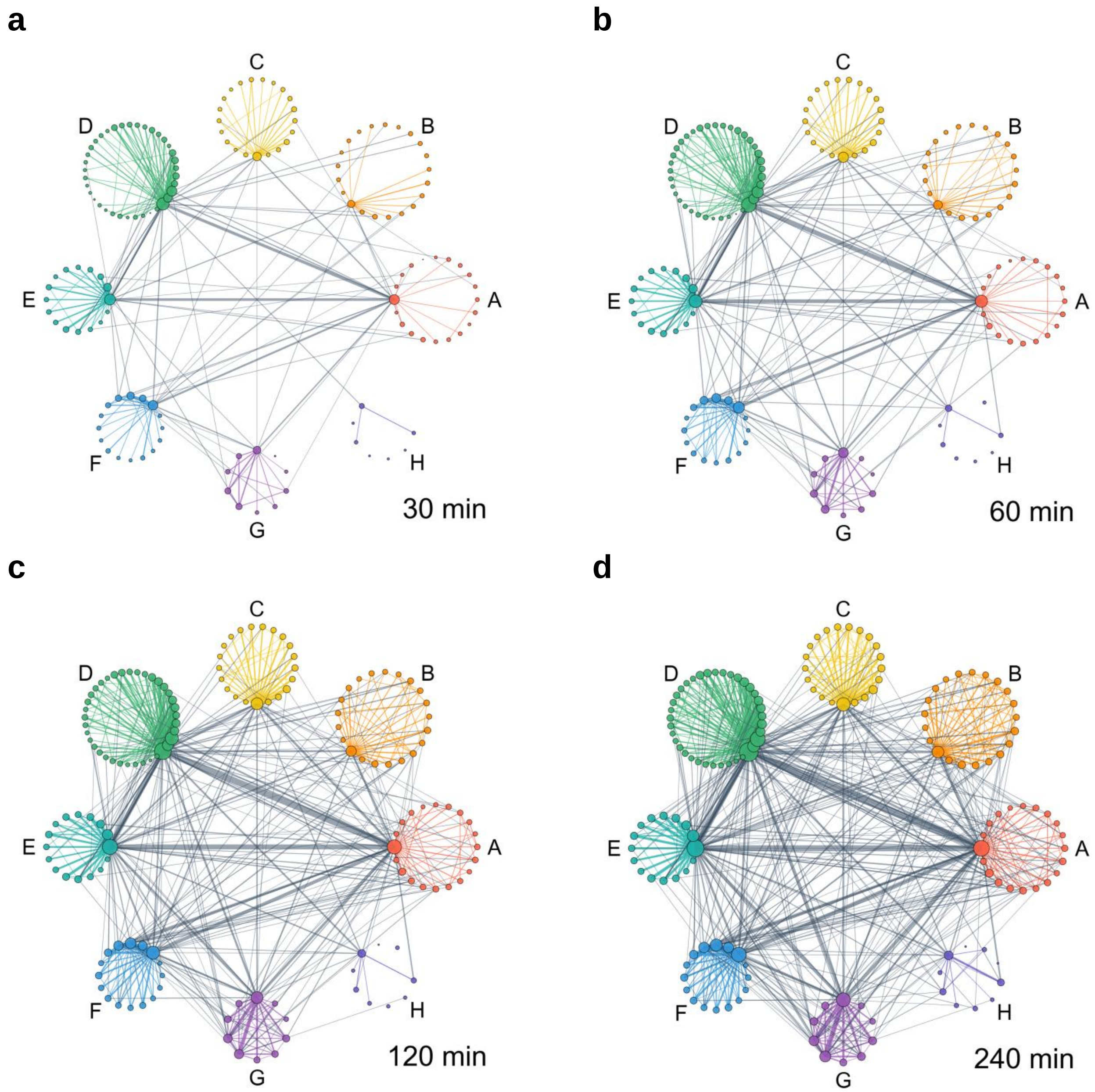

Figure S3. Spatial-temporal evolution of important tie networks across communities (first outgoing calls). Each graph shows network activation after the first 30 minutes (**a**), 60 minutes (**b**), 120 minutes (**c**), and 240 minutes (**d**). Edges are normalized, threshold value is 1%. Geographical coding (circles A to H) corresponds with Table S2. The largest node in each circle represents the county seat (while largest node in circle A is the metropolitan city); other nodes represent towns and villages in each county.

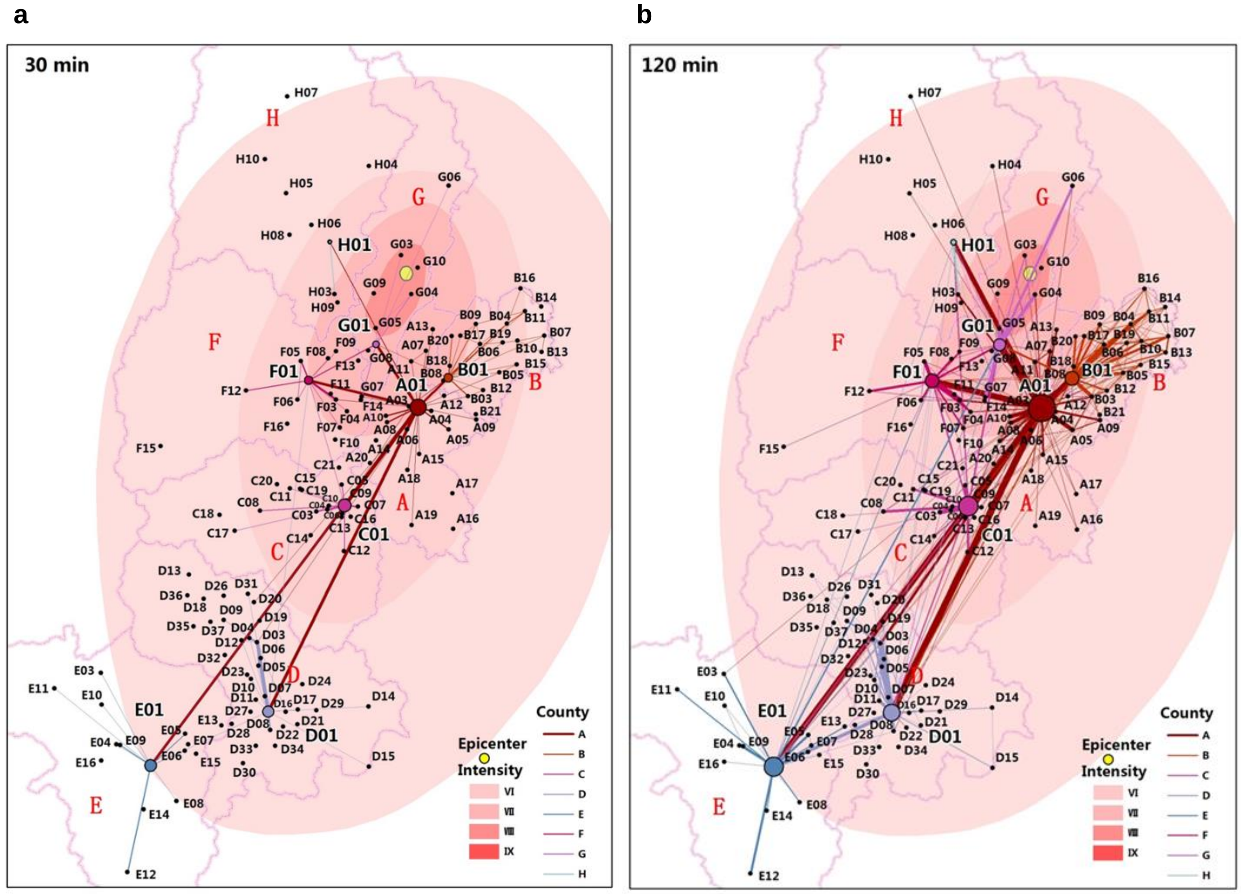

Figure S4. First outgoing calls in Ya’an occurring within 30 minutes (**a**) and 120 minutes (**b**) of the earthquake, transposed onto map of Ya’an. Edge width is proportional to the square root of the number of contacts between the two nodes; edge threshold set to 5. Geographical coding (circles A to H) corresponds with Table S2. The 01 node under each geographical code represents the county seat (while the node A01 is the metropolitan city); other numbered nodes represent towns and villages in each county.

The location of important ties did not conform to the assumptions of distance-based gravity models (Barbosa et al. 2018). Rather, the important tie macro-network had an overlapping and hierarchical community structure (Moody and White 2013), which reflected Ya’an’s socio-economic and administrative structure which also affected migration patterns and economic wellbeing.

Overall, we observed: 1) Localization and clustering effects: First outgoing calls were mainly directed within each county, 2) Centralization effects: the prefecture level city received the highest percentages of calls from all areas, and 3) Epicenter effects: the epicenter Lushan (G) received a relatively higher percentage of calls over the first few hours (Fig. 3 in main text, S4).

2.3 Local versus non-local communications

We found that in the first 10 days after the earthquake, only 13.1% of first outgoing calls (i.e., to important ties) from Ya’an residents were to regions outside of Ya’an prefecture, and thus focused our analysis on the Ya’an network. The majority of first outgoing calls were directed to Ya’an (86.09%). 37.91% of the total incoming calls were from outside of Ya’an, but only 13.91% of the total outgoing calls were made to people outside of Ya’an.

3 Supporting Figures

3.1 Supporting Figures for Figures in Main Text

The following figures support the model-free analysis and figures in the main text.

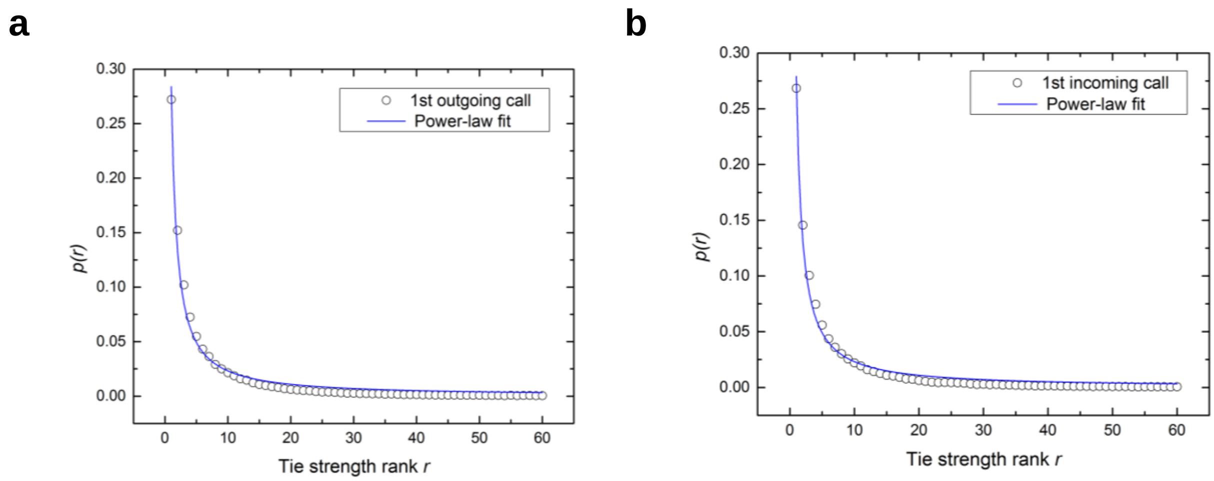

Figure S5. Tie strength and tie importance. The probability distribution $p(r)$ of important ties over the ranking r of tie strength (based on communications frequency during normal times) follows a power law function, $p(r) \sim r^{-\lambda}$. The distributions of first outgoing calls (**a**) and first incoming calls (**b**) are fitted with exponents λ = 1.089 and 1.081 (estimated using the maximum-likelihood method), with adjusted-R^2 values of 0.984 and 0.985, respectively. This distribution suggests that important ties and most frequent ties are separate, albeit correlated, measures of tie strength.

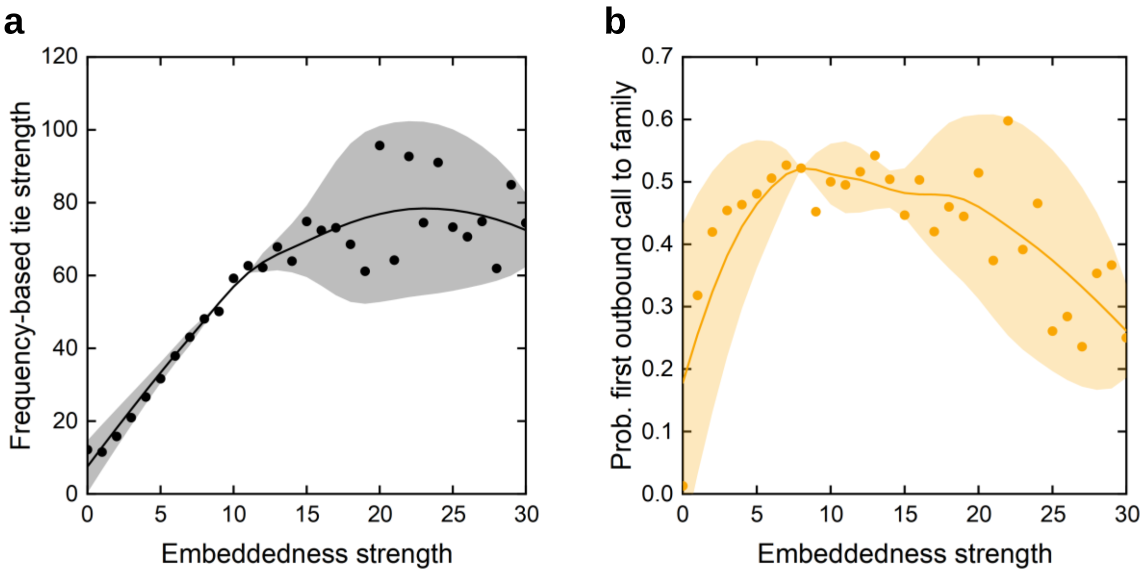

Figure S6. Relationship between embeddedness strength, tie strength. a, Tie strength increases as a function of embeddedness strength (i.e., number of overlapping friends) up to a certain point (~15). **b**, Likelihood that the first outbound call is directed to family has a curvilinear relationship with embeddedness strength, which initially increases but declines after ~7; the strongest predictor is simply the binary variable of whether family plan ties are embedded or unembedded. Embedded family ties are more likely to receive first outbound calls (Pr = 0.053 vs. 0.440, p <.001).

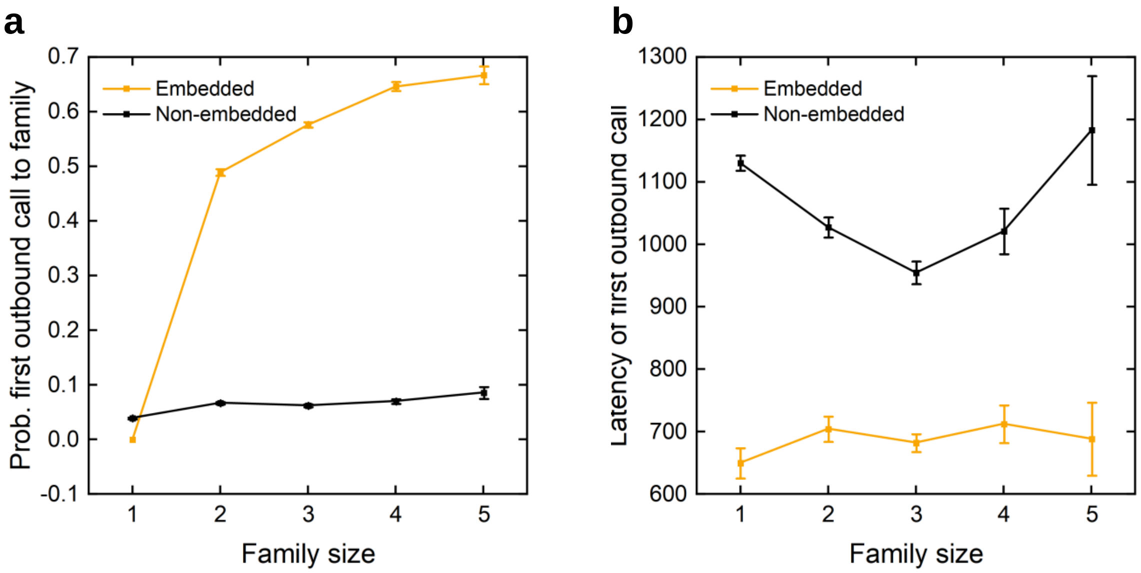

Figure S7. Family size effect on predicting if important tie is family. a, The likelihood that first outgoing call is to family increases with family size when the tie is embedded; this relationship is not apparent when the tie is unembedded. **b**, Embedded ties are activated sooner than unembedded ones; this is independent of family size.

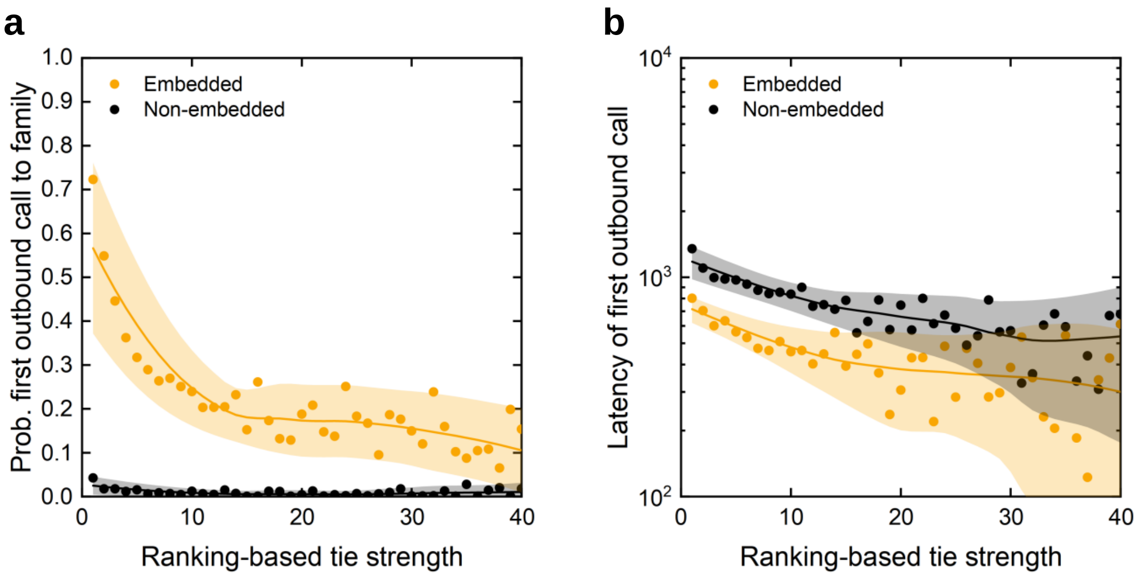

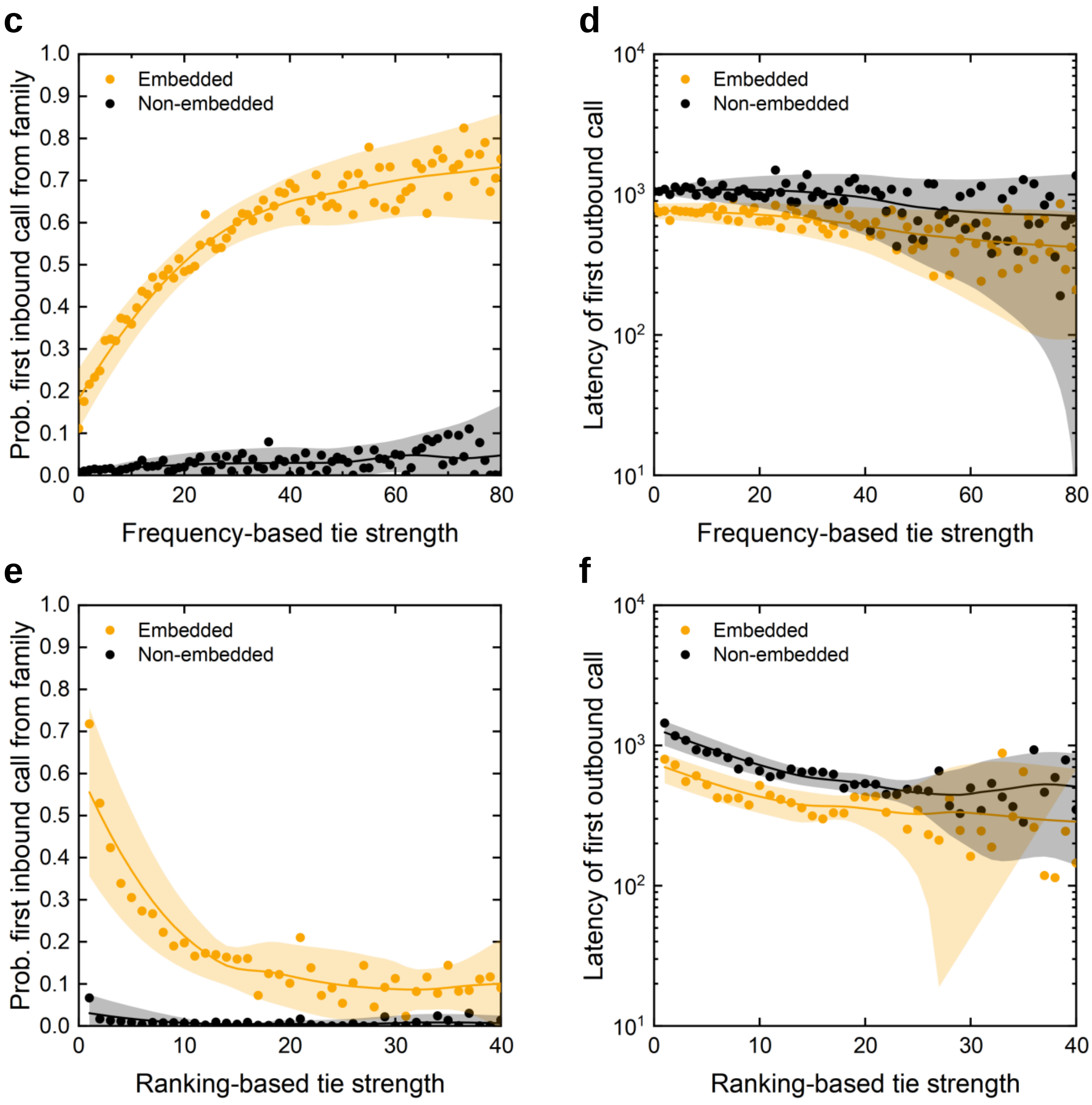

Figure S8. Robustness check using ranking-based measure of tie strength. a-b, We observe analogous patterns for first *outbound* calls when using ranking-based measures of tie strength, as opposed to and tie-strength based measures (seen in main Fig. 1e and f). **c-f**, The pattern of results for first incoming calls is analogous to the results for first outgoing calls. Again, tie strength and tie ranking are only predictive of likelihood that first incoming call is from family when ties are embedded. However, both frequency-based and ranking-based measures of tie strength are predictive of latency of first incoming call; although embedded ties typically receive calls faster.

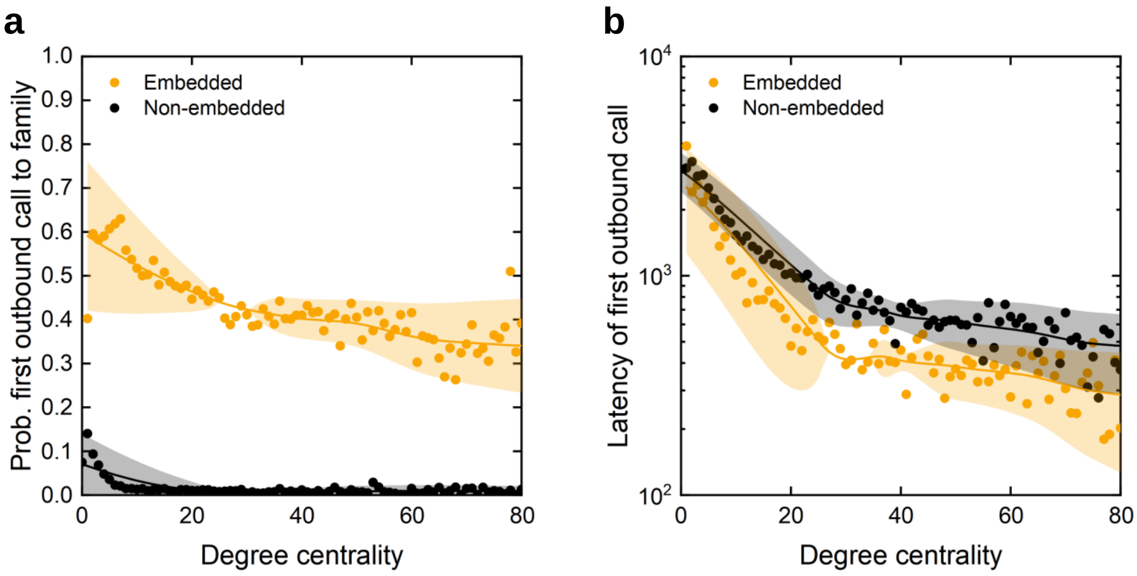

Figure S9. Embeddedness and degree centrality of important ties. a, The likelihood that the first outgoing call is directed to family is a decreasing function of degree centrality when the tie is embedded, and embeddedness plays a larger role when network size (i.e., degree centrality) is relatively small. **b**, The activation latency of important ties can be predicted by both degree centrality and embeddedness.

4.2 Incoming vs. outgoing communications

Overall, the temporal occurrence of first incoming and outgoing communications were relatively symmetric. This is expected considering that most meaningful social relationships are usually characterized by a high degree of reciprocity. We found that the alters who initiated first incoming calls had a similar overall distribution in tie strength (as measured by frequency of communications prior to the earthquake) as alters who received first outgoing calls (important ties), following a power-law function, $p(r) \sim r^{-\beta}$, with a scaling parameter $\beta = 1.081$. The tie strength rankings for the alters of the first outgoing and incoming calls were relatively consistent over time; Spearman's rho correlations of tie strength rankings prior- and post- earthquake were $r_s = 0.730$ ($p < 0.001$) for first outgoing communications and $r_s = 0.736$ ($p < 0.001$) for the first incoming communications.

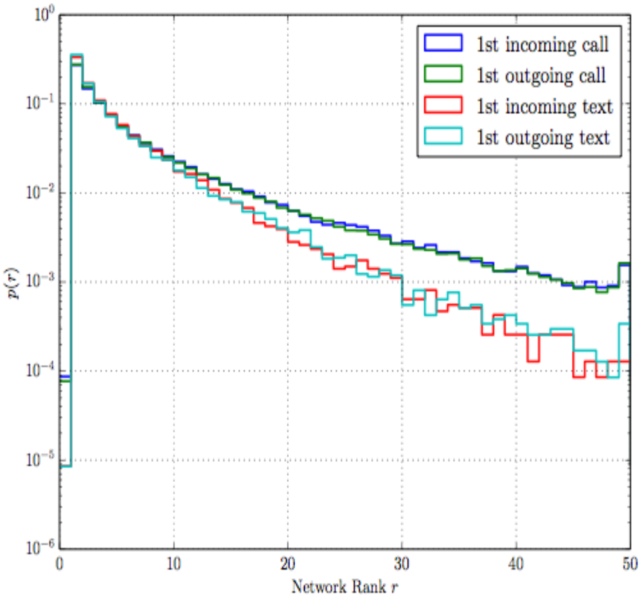

Figure S10. Tie strength ranking of first incoming versus outgoing communications

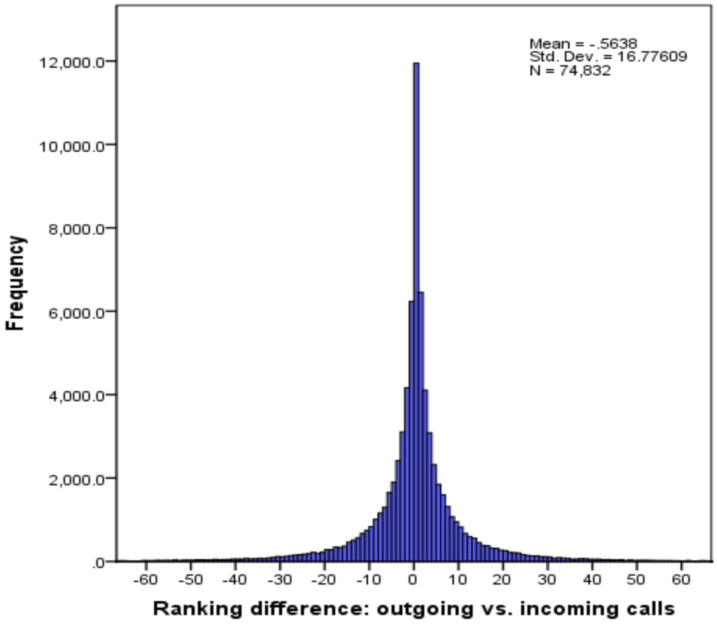

Figure S11. Tie strength ranking of first incoming versus outgoing call. Although the distributions of incoming and outgoing calls were symmetric, the relationship between the two are not perfectly consistent, with a Spearman's rank correlation of $r_S = 0.227$ ($p < 0.001$, Figure S11). Only 15.8% of the first outgoing and incoming contacts had the same ranking (i.e., exhibited perfect reciprocity) and 47.6%

had small differences in tie strength ranking (ranking differences of within ±5). 36.4% had big differences in tie strength rankings (ranking differences of ±5 or greater).

4 Supporting Tables and Models

The following tables are baseline models corresponding to the main models and tables in the main text and robustness checks (see Methods and Materials for details).

4.1 Baseline models for response latency.

The baseline models explore the relationship between social network activation and tie strength and embeddedness without modelling the impact of the earthquake. We conduct robustness checks using a rank percentile measure of tie strength (Model 1.1.2), selecting only for roaming users (1.1.3), and for the second through fourth outgoing calls (1.1.4-1.1.6).

4.2 Three-way-interaction models including earthquake intensity

These models test the interaction between the earthquake intensity and tie strength and embeddedness. This corresponds to Table 1 (i.e., Model 1.2) in the main text. We conduct robustness checks selecting only for roaming users (1.2.2), and for the second through fourth outgoing calls (1.2.3-1.2.5).

4.3 Reciprocity models

We provide a baseline model for the relationship between reciprocity latency and tie strength and embeddedness without modelling the impact of the earthquake (Model 2.1). This corresponds to Table 2 (Model 2.2) in the main text.

4.4 Predicting if important tie is a family plan member

Model 3 tests whether tie strength and embeddedness can predict who the important tie is, and in particular, whether they are a family member (operationalized by family plan membership). Model 3.1 is a baseline model without the earthquake interaction effect. Model 3.2 is included in the main text as Table 3. Model 3.3 is a robustness check selecting only for customers who subscribe to internet services. Model 3.4 selects only roaming users.

4.5 Robustness checks using non-WeChat users

Here, as another robustness check to rule out the WeChat adoption issue (which we discussed earlier in Section 1), we repeat the empirical analyses after excluding WeChat users in our data (subscribers who had previously used WeChat before the earthquake). This additional analysis has yielded consistent results for all three main models; social network activation latency (Table S17), immediate reciprocity (Table S18), and family plan tie activation prediction (Table S19).

4.6 Decision tree analysis

To provide converging evidence using a non-linear model, we use a decision tree model to study the predictive capacity of tie strength and embeddedness. Decision trees are a non-parametric supervised learning method used for classification and regression, and can help discover interactions among independent variables, in which case variables would appear together in a traversal path (since the condition of a child node is dependent of the parent node).

We constructed decision tree models for each of the three main dependent variables: 1) activation latency (Fig. S12), 2) immediate reciprocity (Fig. S13), and family tie activation (Fig. S14). We start each tree without tie strength and embeddedness, then sequentially add tie strength and embeddedness to the tree, leading to a complete tree that incorporates both. As shown in Table S20, incorporating tie strength or embeddedness into the tree improves prediction accuracy; the complete tree including both variables has the greatest predictive power. This highlights that the enhanced predictive power from the joint effect of tie strength and embeddedness, as opposed to considering them separately.

The decision trees illustrate the interactive relationship between tie strength and embeddedness. In Model 1 (Figure S12), if tie strength is already strong (e.g., greater than 6.5), a higher level of embeddedness (e.g., greater than 8.5) predicts a slower initial outbound call (76%) instead of a faster one (24%). Likewise, in Model 2 (Figure S13, if the level of embeddedness is higher (e.g., greater than 0.5), a strong tie (e.g., greater than 3.5) predicts lower probability of a reciprocal call (e.g., less than 67%, as opposed to 72% if the tie strength is just slightly greater than 2.5). Finally, in Model 3 (Figure S14), given higher embeddedness (e.g., greater than 0.5) and stronger ties (e.g., greater than 50.5) are associated with lower likelihood of calling family members (e.g., 75% vs. 79% if the tie strength is just slightly greater than 11.5). Overall, the non-linear decision tree analysis provides converging evidence for our regression model results and intuitively illustrates the interactive relationship between tie strength and embeddedness.

4.7 Random forest model

As an additional analysis, we use a random forest model to generate importance scores for the key predictors of interest, namely tie strength and embeddedness, which helps provide greater interpretability for our results. We constructed a random forest of 100 trees for Model 3. As shown in Table S21, the prediction accuracies of this random forest align closely with those derived from a single tree. The scores for variable importance highlight that tie strength and embeddedness are the two most significant predictors: The importance scores are 57.32 (57.25 for a single tree) for tie strength and 29.62 (29.81 for a single tree) for embeddedness. Note that the importance scores are scaled to a cumulative total of 100 for easier comparison. These results provide additional support to the notion that tie strength and embeddedness should be considered jointly, as opposed to separately, in our study context.

Table S3. Baseline Model 1.1 (2-way interaction only) for social network activation latency

Dependent Variable = *Latency of 1st outgoing call (hours)*	Coef.	Robust S.E.	z	P>|z|	
**Tie strength of important tie**	**-0.0012**	**<0.001**	**-188.13**	**<0.001**	***
**Embeddedness (OP) of important tie**	**-0.0444**	**<0.001**	**-642.95**	**<0.001**	***
**Tie strength*Embeddedness (OP) of important tie**	**0.0005**	**<0.001**	**667.10**	**<0.001**	***
Important tie is family dummy	-0.1470	<0.001	-402.91	<0.001	***
Family plan size (1 to 5)	0.0060	<0.001	53.13	<0.001	***
Degree centrality of ego	0.0003	<0.001	67.74	<0.001	***
Total call frequency of ego	-0.0037	<0.001	-3005.41	<0.001	***
Total text frequency of ego	0.0003	<0.001	308.56	<0.001	***
Internet usage frequency of ego	<0.0001	<0.001	22.20	<0.001	***
Total WeChat usage frequency of ego	-0.0002	<0.001	-92.46	<0.001	***
Total usage frequency of other instant messaging of ego	-0.0007	<0.001	-784.37	<0.001	***
Smartphone dummy (1 = smartphone user)	-0.1170	<0.001	-477.18	<0.001	***
Roaming dummy (1 = traveling outside of prefecture)	-0.5707	<0.001	-1319.72	<0.001	***
Rural dummy (1 = rural)	0.0325	<0.001	86.42	<0.001	***
Damage dummy (1 = cell towers damaged)	-0.0912	0.003	-30.95	<0.001	***
Constant	8.5710	0.003	3224.19	<0.001	***
Pseudo R squared: 0.2172					
Number of obs = 89,907					

All latency models (Tables S6-15) use an exponential mean model (Poisson regression). In all models, communications variables are average monthly data from 4 weeks before the earthquake. Fixed effects for 159 counties are included.

Table S4. Baseline Model 1.1.2 with tie strength operationalized as *rank percentile*

Dependent Variable = *Latency of 1st outgoing call (hours)*	Coef.	Robust S.E.	z	P>|z|	
**Tie strength of important tie**	**-0.2412**	**0.001**	**-406.42**	**<0.001**	***
**Embeddedness (OP) of important tie**	**-0.1318**	**<0.001**	**-420.02**	**<0.001**	***
**Tie strength*Embeddedness (OP) of important tie**	**0.1323**	**<0.001**	**381.15**	**<0.001**	***
Important tie is family dummy	-0.1272	<0.001	-314.33	<0.001	***
Degree centrality of ego	-0.0023	<0.001	-15.45	<0.001	***
Total call frequency of ego	-0.0035	<0.001	-2706.13	<0.001	***
Total text frequency of ego	0.0003	<0.001	249.42	<0.001	***
Internet usage frequency of ego	<0.0001	<0.001	42.53	<0.001	***
Total WeChat usage frequency of ego	<0.0001	<0.001	-10.31	<0.001	***
Total usage frequency of other instant messaging of ego	-0.0007	<0.001	-634.82	<0.001	***
Smartphone dummy (1 = smartphone user)	-0.1299	<0.001	-417.17	<0.001	***
Roaming dummy (1 = traveling outside of prefecture)	-0.5357	0.001	-984.36	<0.001	***
Rural dummy (1 = rural)	0.0358	<0.001	74.86	<0.001	***
Damage dummy (1 = cell towers damaged)	0.0070	0.004	1.90	0.057	.
Constant	8.7830	0.003	2677.41	<0.001	***
Pseudo R squared: 0.2228					
Number of obs = 65,442[2]					

Rank Percentile Score is calculated as the tie's absolute rank divided by the call network size. Our results are robust after rank transformation.

[2] Smaller sample size due to missing data in calculating ranking cumulative tie strength.

Table S5. Baseline Model 1.1.3 for social network activation latency of *roaming users only*

Dependent Variable = *Latency of 1st outgoing call (hours)*	Coef.	Robust S.E.	z	P>|z|	
**Tie strength of important tie**	**0.0022**	**<0.001**	**101.75**	**<0.001**	***
**Embeddedness (OP) of important tie**	**-0.1097**	**<0.001**	**-365.48**	**<0.001**	***
**Tie strength*Embeddedness of important tie**	**0.0005**	**<0.001**	**131.29**	**<0.001**	***
Important tie is family dummy	-0.3063	0.001	-256.81	<0.001	***
Family plan size (1 to 5)	-0.0020	<0.001	-4.60	<0.001	***
Degree centrality of ego	-0.0084	<0.001	-212.71	<0.001	***
Total call frequency of ego	-0.0017	<0.001	-271.39	<0.001	***
Total text frequency of ego	0.0003	<0.001	123.14	<0.001	***
Internet usage frequency of ego	-0.0001	<0.001	-58.52	<0.001	***
Total WeChat usage frequency of ego	0.0001	<0.001	17.36	<0.001	***
Total usage frequency of other instant messaging of ego	-0.0005	<0.001	-187.78	<0.001	***
Smartphone dummy (1 = smartphone user)	-0.2115	0.001	-233.81	<0.001	***
Rural dummy (1 = rural)	-0.0891	0.002	-55.97	<0.001	***
Damage dummy (1 = cell towers damaged)	0.8914	0.027	33.45	<0.001	***
Constant	6.6990	0.026	255.46	<0.001	***
Pseudo R squared: 0.1566					
Number of obs = 10,712					

Table S6. Baseline Model 1.1.4 for latency of *second* outgoing call (2-way interaction only)

Dependent Variable = *Latency of second outgoing call (hours)*	Coef.	Robust S.E.	z	P>|z|	
**Tie strength of important tie**	**-0.0014**	**<0.001**	**-283.53**	**<0.001**	***
**Embeddedness (OP) of important tie**	**-0.0338**	**<0.001**	**-657.65**	**<0.001**	***
**Tie strength*Embeddedness of important tie**	**0.0002**	**<0.001**	**327.99**	**<0.001**	***
Family plan size (1 to 5)	-0.0109	<0.001	-121.84	<0.001	***
Degree centrality of ego	0.0002	<0.001	48.08	<0.001	***
Total call frequency of ego	-0.0036	<0.001	-3453.33	<0.001	***
Total text frequency of ego	0.0002	<0.001	195.48	<0.001	***
Internet usage frequency of ego	<0.0001	<0.001	4.25	<0.001	***
Total WeChat usage frequency of ego	-0.0001	<0.001	-49.29	<0.001	***
Total usage frequency of other instant messaging of ego	-0.0005	<0.001	-751.55	<0.001	***
Smartphone dummy (1 = smartphone user)	-0.1526	<0.001	-762.38	<0.001	***
Roaming dummy (1 = traveling outside of prefecture)	-0.3170	<0.001	-1010.04	<0.001	***
Rural dummy (1 = rural)	0.0473	<0.001	153.14	<0.001	***
Damage dummy (1 = cell towers damaged)	-0.0599	0.003	-22.83	<0.001	***
Constant	8.5710	0.003	3224.19	<0.001	***
Pseudo R squared: 0.2247					
Number of obs = 88,490					

Table S7. Baseline Model 1.1.5 for latency of *third* outgoing call (2-way interaction only)

Dependent Variable = *Latency of third outgoing call (hours)*	Coef.	Robust S.E.	z	P>|z|	
**Tie strength of important tie**	**0.0008**	**<0.001**	**189.14**	**<0.001**	***
**Embeddedness (OP) of important tie**	**-0.0276**	**<0.001**	**-587.95**	**<0.001**	***
**Tie strength*Embeddedness of important tie**	**0.0002**	**<0.001**	**296.24**	**<0.001**	***
Family plan size (1 to 5)	-0.0160	<0.001	-203.43	<0.001	***
Degree centrality of ego	0.0005	<0.001	130.01	<0.001	***
Total call frequency of ego	-0.0036	<0.001	-4020.61	<0.001	***
Total text frequency of ego	0.0001	<0.001	97.76	<0.001	***
Internet usage frequency of ego	<0.0001	<0.001	-40.66	<0.001	***
Total WeChat usage frequency of ego	-0.0001	<0.001	-44.15	<0.001	***
Total usage frequency of other instant messaging of ego	-0.0004	<0.001	-723.94	<0.001	***
Smartphone dummy (1 = smartphone user)	-0.1587	<0.001	-906.75	<0.001	***
Roaming dummy (1 = traveling outside of prefecture)	-0.1814	<0.001	-695.97	<0.001	***
Rural dummy (1 = rural)	0.0465	<0.001	171.57	<0.001	***
Damage dummy (1 = cell towers damaged)	0.0278	0.003	10.68	<0.001	***
Constant	8.8330	0.002	3710.88	<0.001	***
Pseudo R squared: 0.2386					
Number of obs = 87,091					

Table S8. Baseline Model 1.1.6 for latency of *fourth* outgoing call (2-way interaction only)

Dependent Variable = *Latency of fourth outgoing call (hours)*	Coef.	Robust S.E.	z	P>|z|	
**Tie strength of important tie**	**0.0006**	**<0.001**	**165.81**	**<0.001**	***
**Embeddedness (OP) of important tie**	**-0.0184**	**<0.001**	**-434.88**	**<0.001**	***
**Tie strength*Embeddedness of important tie**	**0.0001**	**<0.001**	**207.15**	**<0.001**	***
Family plan size (1 to 5)	-0.0208	<0.001	-289.07	<0.001	***
Degree centrality of ego	0.0003	<0.001	90.21	<0.001	***
Total call frequency of ego	-0.0034	<0.001	-4032.45	<0.001	***
Total text frequency of ego	<0.0001	<0.001	62.91	<0.001	***
Internet usage frequency of ego	<0.0001	<0.001	-20.16	<0.001	***
Total WeChat usage frequency of ego	-0.0002	<0.001	-106.32	<0.001	***
Total usage frequency of other instant messaging of ego	-0.0003	<0.001	-718.36	<0.001	***
Smartphone dummy (1 = smartphone user)	-0.1561	<0.001	-982.39	<0.001	***
Roaming dummy (1 = traveling outside of prefecture)	-0.1170	<0.001	-505.58	<0.001	***
Rural dummy (1 = rural)	0.0422	<0.001	171.33	<0.001	***
Damage dummy (1 = cell towers damaged)	0.0425	0.003	16.70	<0.001	***
Constant	8.8980	0.002	3819.44	<0.001	***
Pseudo R squared: 0.2513					
Number of obs = 85,682					

Table S9. Model 1.2.2 for social network activation latency of *roaming users only*

Dependent Variable = *Latency of first outgoing call (hours)*	Coef.	Robust S.E.	z	P>|z|	
Tie strength of important tie	0.0029	<0.001	108.79	<0.001	***
Embeddedness (OP) of important tie	-0.1141	<0.001	-285.14	<0.001	***
Earthquake intensity dummy (1 = severe)	1.8420	0.030	62.05	<0.001	***
Tie strength*Embeddedness (OP) of important tie	0.0003	<0.001	49.34	<0.001	***
Tie strength*Earthquake intensity dummy	-0.0016	<0.001	-38.89	<0.001	***
Embeddedness (OP) of important tie*Earthquake intensity dummy	0.0182	0.001	31.14	<0.001	***
**Tie strength*Embeddedness (OP) of important tie*Earthquake intensity dummy**	**0.0002**	**<0.001**	**32.77**	**<0.001**	***
Important tie is family dummy	-0.3043	0.001	-254.89	<0.001	***
Family plan size (1 to 5)	-0.0022	<0.001	-5.09	<0.001	***
Degree centrality of ego	-0.0084	<0.001	-213.19	<0.001	***
Total call frequency of ego	-0.0017	<0.001	-270.70	<0.001	***
Smartphone dummy (1 = smartphone user)	0.0003	<0.001	122.63	<0.001	***
Internet usage frequency of ego	-0.0001	<0.001	-58.95	<0.001	***
Total WeChat usage frequency of ego	0.0001	<0.001	16.75	<0.001	***
Total usage frequency of other instant messaging of ego	-0.0005	<0.001	-187.67	<0.001	***
Smartphone dummy (1 = smartphone user)	-0.2112	0.001	-233.45	<0.001	***
Rural dummy (1 = rural)	-0.0889	0.002	-55.85	<0.001	***
Damage dummy (1 = cell towers damaged)	-0.9578	0.015	-65.20	<0.001	***
Tie strength of important tie	0.0029	<0.001	108.79	<0.001	***
Constant	6.7060	0.026	255.70	<0.001	***
Pseudo R squared: 0.1569					
Number of obs = 10,712					

Communications variables are average monthly data from 4 weeks before the earthquake. Fixed effects for 159 counties are included.

The above analysis is the same as that for Table 1 (Model 2.1), except it only includes customers who were on roaming, i.e., out of town during the earthquake. Most germane to our hypotheses, the 3-way interaction effect is consistent with the main model (Table 1). However, here, tie strength has a positive main effect and a negative two-way interaction with earthquake intensity. The differences in coefficients are difficult to interpret since they might reflect different social dynamics for those who are roaming frequently (e.g. if they have different relationships with their important ties), or that the roaming sample has different social characteristics, e.g., may be migrant workers.

Table S10. Model 1.2.3 for social network activation latency of *second* outgoing call

Dependent Variable = *Latency of second outgoing call (hours)*	Coef.	Robust S.E.	z	P>|z|	
Tie strength of important tie	-0.0020	<0.001	-325.91	<0.001	***
Embeddedness (OP) of important tie	-0.0307	<0.001	-456.86	<0.001	***
Earthquake intensity dummy (1 = severe)	0.0584	0.004	16.44	<0.001	***
Tie strength*Embeddedness (OP) of important tie	0.0002	<0.001	230.67	<0.001	***
Tie strength*Earthquake intensity dummy	0.0018	<0.001	180.36	<0.001	***
Embeddedness (OP) of important tie*Earthquake intensity dummy	-0.0071	<0.001	-69.54	<0.001	***
**Tie strength*Embeddedness (OP) of important tie*Earthquake intensity dummy**	**<0.0001**	**<0.001**	**5.79**	**<0.001**	***
Family plan size (1 to 5)	-0.0110	<0.001	-122.67	<0.001	***
Degree centrality of ego	0.0002	<0.001	47.27	<0.001	***
Total call frequency of ego	-0.0036	<0.001	-3448.94	<0.001	***
Smartphone dummy (1 = smartphone user)	0.0002	<0.001	196.38	<0.001	***
Internet usage frequency of ego	<0.0001	<0.001	4.19	<0.001	***
Total WeChat usage frequency of ego	-0.0001	<0.001	-48.65	<0.001	***
Total usage frequency of other instant messaging of ego	-0.0005	<0.001	-752.20	<0.001	***
Smartphone dummy (1 = smartphone user)	-0.1523	<0.001	-760.97	<0.001	***
Roaming dummy (1 = traveling outside of prefecture)	-0.3170	<0.001	-1010.20	<0.001	***
Rural dummy (1 = rural)	0.0476	<0.001	154.06	<0.001	***
Damage dummy (1 = cell towers damaged)	-0.1262	0.003	-43.71	<0.001	***
Fixed Effects (159 Counties)	Yes				
Constant	8.8090	0.002	3711.97	<0.001	***
Pseudo R squared: 0.2249					

Number of obs = 88,490

The above analysis is the same as that for Table 1, except that the dependent variable is the latency of the second outgoing voice call (as opposed to 1st outgoing voice call). We observe the same significant, positive three-way interaction in all models.

Table S11. Model 1.2.4 for social network activation latency of *third* outgoing call

Dependent Variable = *Latency of third outgoing call (hours)*	Coef.	Robust S.E.	z	P>|z|	
Tie strength of important tie	0.0001	<0.001	13.34	<0.001	***
Embeddedness (OP) of important tie	-0.0263	<0.001	-430.46	<0.001	***
Earthquake intensity dummy (1 = severe)	0.1896	0.004	54.02	<0.001	***
Tie strength*Embeddedness (OP) of important tie	0.0002	<0.001	175.34	<0.001	***
Tie strength*Earthquake intensity dummy	0.0021	<0.001	246.97	<0.001	***
Embeddedness (OP) of important tie*Earthquake intensity dummy	-0.0017	<0.001	-18.60	<0.001	***
**Tie strength*Embeddedness (OP) of important tie*Earthquake intensity dummy**	**<0.0001**	**<0.001**	**37.37**	**<0.001**	***
Family plan size (1 to 5)	-0.0161	<0.001	-204.12	<0.001	***
Degree centrality of ego	0.0005	<0.001	129.87	<0.001	***
Total call frequency of ego	-0.0036	<0.001	-4016.87	<0.001	***
Smartphone dummy (1 = smartphone user)	0.0001	<0.001	97.47	<0.001	***
Internet usage frequency of ego	<0.0001	<0.001	-40.95	<0.001	***
Total WeChat usage frequency of ego	-0.0001	<0.001	-42.85	<0.001	***
Total usage frequency of other instant messaging of ego	-0.0004	<0.001	-725.44	<0.001	***
Smartphone dummy (1 = smartphone user)	-0.1587	<0.001	-906.62	<0.001	***
Roaming dummy (1 = traveling outside of prefecture)	-0.1820	<0.001	-698.17	<0.001	***
Rural dummy (1 = rural)	0.0464	<0.001	171.26	<0.001	***
Damage dummy (1 = cell towers damaged)	-0.1742	0.003	-62.11	<0.001	***
Fixed Effects (159 Counties)	Yes				
Constant	8.8370	0.002	3712.41	<0.001	***
Pseudo R squared: 0.2390					
Number of obs = 87,091					

Poisson regression. Communications variables are average monthly data from 4 weeks before the earthquake. Fixed effects for 159 counties are included.

Table S12. Model 1.2.5 for social network activation latency of *fourth* outgoing call

Dependent Variable = *Latency of fourth outgoing call (hours)*	Coef.	Robust S.E.	z	P>|z|	
Tie strength of important tie	-0.0003	<0.001	-53.76	<0.001	***
Embeddedness (OP) of important tie	-0.0153	<0.001	-287.61	<0.001	***
Earthquake intensity dummy (1 = severe)	0.2075	0.003	61.18	<0.001	***
Tie strength*Embeddedness (OP) of important tie	0.0001	<0.001	148.46	<0.001	***
Tie strength*Earthquake intensity dummy	0.0024	<0.001	323.51	<0.001	***
Embeddedness (OP) of important tie*Earthquake intensity dummy	-0.0075	<0.001	-88.50	<0.001	***
**Tie strength*Embeddedness (OP) of important tie*Earthquake intensity dummy**	**<0.0001**	**<0.001**	**21.56**	**<0.001**	***
Family plan size (1 to 5)	-0.0209	<0.001	-290.99	<0.001	***
Degree centrality of ego	0.0003	<0.001	90.61	<0.001	***
Total call frequency of ego	-0.0034	<0.001	-4026.39	<0.001	***
Smartphone dummy (1 = smartphone user)	<0.0001	<0.001	60.11	<0.001	***
Internet usage frequency of ego	<0.0001	<0.001	-17.28	<0.001	***
Total WeChat usage frequency of ego	-0.0002	<0.001	-106.89	<0.001	***
Total usage frequency of other instant messaging of ego	-0.0003	<0.001	-719.25	<0.001	***
Smartphone dummy (1 = smartphone user)	-0.1562	<0.001	-983.22	<0.001	***
Roaming dummy (1 = traveling outside of prefecture)	-0.1172	<0.001	-506.42	<0.001	***
Rural dummy (1 = rural)	0.0425	<0.001	172.68	<0.001	***
Damage dummy (1 = cell towers damaged)	-0.1736	0.003	-64.73	<0.001	***
Fixed Effects (159 Counties)	Yes				
Constant	8.9010	0.002	3820.57	<0.001	***
Pseudo R squared: 0.2518					

Number of obs = 85,682

Poisson regression. Communications variables are average monthly data from 4 weeks before the earthquake. Fixed effects for 159 counties are included.

*One might note that the sample size was only slightly smaller for Table S7; i.e., 95% of customers made at least four outgoing calls in our observation period. Our results are robust for outgoing calls 1 through 4.

Table S13. Baseline Model 2.1 for reciprocity (2-way interaction only)

Dependent Variable = *p*(reciprocal call)	Coef.	Robust S.E.	z	P>|z|	
**Tie strength of important tie**	**0.1643**	**0.005**	**31.34**	**<0.001**	***
**Embeddedness (OP) of important tie**	**0.3329**	**0.019**	**17.18**	**<0.001**	***
**Tie strength*Embeddedness (OP) of important tie**	**-0.0334**	**0.008**	**-4.05**	**<0.001**	***
Important tie is family dummy	0.4755	0.016	29.08	<0.001	***
Family plan size (1 to 5)	-0.0560	0.006	-9.39	<0.001	***
Degree centrality of ego	-0.0033	<0.001	-8.61	<0.001	***
Total call frequency of ego	-0.0003	<0.001	-5.44	<0.001	***
Total text frequency of ego	-0.0001	<0.001	-1.27	0.203	
Internet usage frequency of ego	<0.0001	<0.001	-0.09	0.926	
Total WeChat usage frequency of ego	-0.0001	<0.001	-0.73	0.464	
Total usage frequency of other instant messaging of ego	<0.0001	<0.001	-0.62	0.536	
Smartphone dummy (1 = smartphone user)	-0.0184	0.012	-1.51	0.131	
Roaming dummy (1 = traveling outside of prefecture)	0.0744	0.017	4.45	<0.001	***
Rural dummy (1 = rural)	0.0212	0.020	1.06	0.290	
Damage dummy (1 = cell towers damaged)	-0.4773	0.237	-2.01	0.044	*
Constant	-0.9413	0.206	-4.57	<0.001	***
Pseudo R squared: 0.0914					
Number of obs = 91,839					

Probit model. Communications variables are average monthly data from 4 weeks before the earthquake. Fixed effects for 159 counties are included.

To address the potential quasi-complete separation problem between the dependent variable and these two key independent variables, we categorize tie strength into five ordered intervals which contains similar amount of data points, which are 0, [1,10], [11,20], [21,30], 31 or above, and categorize embeddedness into 0 and 1 (when embeddedness is larger than 0),

Table S14. Baseline Model 3.1 for predicting if first outgoing call is to family (2-way interaction only)

Dependent Variable = *p*(important tie is family plan member)	Coef.	Robust S.E.	z	P>|z|	
**Tie strength of important tie**	**-0.1112**	**0.010**	**-10.61**	**<0.001**	***
**Embeddedness (OP) of important tie**	**0.6581**	**0.022**	**30.05**	**<0.001**	***
**Tie strength*Embeddedness (OP) of important tie**	**0.5472**	**0.012**	**44.27**	**<0.001**	***
Family plan size (1 to 5)	0.1600	0.010	16.75	<0.001	***
Degree centrality of ego	-0.0004	<0.001	-1.08	0.280	
Total call frequency of ego	-0.0009	<0.001	-13.87	<0.001	***
Total text frequency of ego	-0.0002	<0.001	-2.75	0.006	**
Internet usage frequency of ego	<0.0001	<0.001	-0.49	0.623	
Total WeChat usage frequency of ego	<0.0001	<0.001	-0.04	0.967	
Total usage frequency of other instant messaging of ego	<0.0001	<0.001	-1.04	0.297	
Smartphone dummy (1 = smartphone user)	-0.0798	0.016	-5.13	<0.001	***
Roaming dummy (1 = traveling outside of prefecture)	0.2644	0.020	12.96	<0.001	***
Rural dummy (1 = rural)	0.0524	0.027	1.97	0.049	*
Damage dummy (1 = cell towers damaged)	-0.6770	0.371	-1.83	0.068	.
Constant	-1.4480	0.313	-4.62	<0.001	***
Pseudo R squared: 0.3763					
Number of obs = 54,857					

Probit model. Fixed effects for 159 counties are included. Family plan only (family size 2 and greater)

Table S15. Model 3.1 for predicting if first outgoing call is to family selecting only full-service customers

Dependent Variable = *p*(important tie is a family member)	Coef.	Robust S.E.	z	P>|z|	
Tie strength of important tie	0.0136	<0.001	15.22	<0.001	***
Embeddedness (OP) of important tie	0.1185	0.008	14.54	<0.001	***
Earthquake intensity dummy (1 = severe)	>10000	>1000	0.69	0.490	
Tie strength*Embeddedness (OP) of important tie	-0.0005	<0.001	-4.83	<0.001	***
Tie strength*Earthquake intensity dummy	0.0013	0.001	0.93	0.355	
Embeddedness (OP) of important tie*Earthquake intensity dummy	-0.0825	0.011	-7.36	<0.001	***
**Tie strength*Embeddedness (OP) of important tie*Earthquake intensity dummy**	**0.0008**	**<0.001**	**4.19**	**<0.001**	***
Degree centrality of ego	0.0002	<0.001	0.18	0.858	
Total call frequency of ego	-0.0013	<0.001	-8.56	<0.001	***
Smartphone dummy (1 = smartphone user)	-0.0002	<0.001	-1.97	0.049	*
Internet usage frequency of ego	<0.0001	<0.001	0.73	0.466	
Total WeChat usage frequency of ego	<0.0001	<0.001	-0.11	0.915	
Total usage frequency of other instant messaging of ego	<0.0001	<0.001	-0.65	0.519	
Smartphone dummy (1 = smartphone user)	-0.0165	0.066	-0.25	0.803	
Roaming dummy (1 = traveling outside of prefecture)	0.3263	0.047	6.93	<0.001	***
Rural dummy (1 = rural)	0.0678	0.069	0.98	0.329	
Damage dummy (1 = cell towers damaged)	<0.0001	>1000	-0.69	0.490	
Constant	-0.6880	0.645	-1.07	0.286	
Pseudo R squared: 0.2330					
Number of obs = 7,501					

Probit model. Fixed effects for 159 counties are included.

(1) Family plan only (family size 3)

(2) We selected only customers who subscribe to both the carrier's basic service (call or text) and telecom's value added service (Internet and app usage) to test if the results are different for high-internet usage customers. We find similar results as Table 3 (Model 3) in the main text, which includes customers who do not have a data plan (e.g., elderly or less technologically adept), which suggests that our results are unlikely to be driven by technological sophistication/ internet access related factors.

Table S16. Model 3.1 for predicting if first outgoing call is to family for *roaming users only*

Dependent Variable = *p*(important tie is a family member)	Coef.	Robust S.E.	z	P>|z|	
Tie strength of important tie	0.0214	0.001	14.48	<0.001	***
Embeddedness (OP) of important tie	0.1204	0.011	10.51	<0.001	***
Earthquake intensity dummy (1 = severe)	<0.0001	>1000	-0.92	0.356	
Tie strength*Embeddedness (OP) of important tie	-0.0014	<0.001	-5.94	<0.001	***
Tie strength*Earthquake intensity dummy	0.0034	0.002	1.46	0.145	
Embeddedness (OP) of important tie*Earthquake intensity dummy	-0.0409	0.017	-2.48	0.013	*
**Tie strength*Embeddedness (OP) of important tie*Earthquake intensity dummy**	0.0008	<0.001	1.91	0.056	.
Family plan size (1 to 5)	0.2209	0.024	9.25	<0.001	***
Degree centrality of ego	-0.0027	0.001	-2.40	0.016	*
Total call frequency of ego	-0.0012	<0.001	-7.00	<0.001	***
Smartphone dummy (1 = smartphone user)	<0.0001	<0.001	0.21	0.836	
Internet usage frequency of ego	<0.0001	<0.001	1.43	0.153	
Total WeChat usage frequency of ego	-0.0001	<0.001	-0.47	0.636	
Total usage frequency of other instant messaging of ego	<0.0001	<0.001	0.14	0.887	
Smartphone dummy (1 = smartphone user)	0.0064	0.038	0.17	0.868	
Rural dummy (1 = rural)	0.0858	0.069	1.25	0.213	
Damage dummy (1 = cell towers damaged)	>10000	>1000	0.92	0.356	
Constant	-1.6810	0.761	-2.21	0.027	*
Pseudo R squared: 0.1717					
Number of obs = 6,558					

Probit model. Fixed effects for 159 counties are included.

(1) Family plan only (family size 2 and greater)

(2) Selecting only customers who were roaming/out of town during the earthquake

Table S17. Impact of tie strength and embeddedness on social network activation latency for Non-WeChat Users

Dependent Variable = *Latency of first outgoing call (hours)*	Coef.	Robust S.E.	z	P>|z|	
Tie strength of important tie	-0.0014	<0.001	-128.06	<0.001	***
Embeddedness (OP) of important tie	-0.0505	<0.001	-421.33	<0.001	***
Earthquake intensity dummy (1 = severe)	0.1123	0.004	25.01	<0.001	***
Tie strength*Embeddedness (OP) of important tie	0.0005	<0.001	299.03	<0.001	***
Tie strength*Earthquake intensity dummy	0.0019	<0.001	111.93	<0.001	***
Embeddedness (OP) of important tie*Earthquake intensity dummy	0.0014	<0.001	7.62	<0.001	***
Tie strength*Embeddedness (OP) of important tie*Earthquake intensity dummy	<0.0001	<0.001	31.27	<0.001	***
Important tie is family dummy	-0.1467	<0.001	-338.12	<0.001	***
Family plan size (1 to 5)	0.0177	<0.001	132.53	<0.001	***
Degree centrality of ego	0.0005	<0.001	159.54	<0.001	***
Total call frequency of ego	-0.0047	<0.001	-2883.43	<0.001	***
Total text frequency of ego	0.0004	<0.001	228.40	<0.001	***
Internet usage frequency of ego	0.0003	<0.001	303.00	<0.001	***
Total usage frequency of other instant messaging of ego	-0.0005	<0.001	-129.64	<0.001	***
Smartphone dummy (1 = smartphone user)	0.0061	<0.001	18.65	<0.001	***
Roaming dummy (1 = traveling outside of prefecture)	-0.6302	<0.001	-1166.91	<0.001	***
Rural dummy (1 = rural)	0.0391	<0.001	85.05	<0.001	***
Damage dummy (1 = cell towers damaged)	-0.1972	0.004	-52.51	<0.001	***
Constant	8.5990	0.003	2981.79	<0.001	***
Pseudo R squared: 0.1938					
Number of obs = 51,501					

Poisson regression. Communications variables are average monthly data from 4 weeks before the earthquake. Fixed effects for 159 counties are included.

Table S18. Impact of tie strength and embeddedness on reciprocity for Non-WeChat Users

Dependent Variable = *p*(reciprocal call)	Coef.	Robust S.E.	z	P>|z|	
Tie strength of important tie	0.1683	0.009	19.68	<0.001	***
Embeddedness (OP) of important tie	0.3423	0.033	10.32	<0.001	***
Earthquake intensity dummy (1 = severe)	-0.1369	0.386	-0.36	0.723	
Tie strength*Embeddedness (OP) of important tie	-0.0408	0.014	-2.92	0.004	**
Tie strength*Earthquake intensity dummy	0.0182	0.014	1.32	0.187	
Embeddedness (OP) of important tie*Earthquake intensity dummy	0.0170	0.051	0.33	0.740	
Tie strength*Embeddedness (OP) of important tie*Earthquake intensity dummy	-0.0347	0.022	-1.59	0.111	
Important tie is family dummy	0.5239	0.022	24.24	<0.001	***
Family plan size (1 to 5)	-0.0530	0.008	-6.81	<0.001	***
Degree centrality of ego	-0.0047	<.0001	-7.66	<0.001	***
Total call frequency of ego	-0.0003	<.0001	-3.70	<0.001	***
Total text frequency of ego	<0.0001	<.0001	0.23	0.819	
Internet usage frequency of ego	<0.0001	<.0001	0.23	0.817	
Total usage frequency of other instant messaging of ego	0.0002	<.0001	1.45	0.147	
Smartphone dummy (1 = smartphone user)	0.0126	0.018	0.72	0.471	
Roaming dummy (1 = traveling outside of prefecture)	0.0720	0.023	3.13	0.002	**
Rural dummy (1 = rural)	0.0083	0.026	0.32	0.752	
Damage dummy (1 = cell towers damaged)	-0.3453	0.337	-1.03	0.305	
Constant	-0.8981	0.233	-3.86	<0.001	***
Pseudo R squared: 0.0937					
Number of obs = 53,097					

Probit model. Communications variables are average monthly data from 4 weeks before the earthquake. Fixed effects for 159 counties are included.

To address the potential quasi-complete separation problem between the dependent variable and these two key independent variables, we categorize tie strength into five ordered intervals which contain similar number of data points, which are 0, [1,10], [11,20], [21,30], 31 or above, and categorize embeddedness as 0 or 1 (when embeddedness is larger than 0).

Table S19. Predicting if first outgoing call after earthquake is to family for Non-WeChat Users

Dependent Variable = *p*(important tie is family plan member)	Coef.	Robust S.E.	z	P>|z|	
Tie strength of important tie	0.0153	<0.001	28.23	<0.001	***
Embeddedness (OP) of important tie	0.1872	0.005	35.97	<0.001	***
Earthquake intensity dummy (1 = severe)	-0.8805	0.639	-1.38	0.168	
Tie strength*Embeddedness of important tie	-0.0008	<0.001	-13.01	<0.001	***
Tie strength*Earthquake intensity dummy	0.0046	<0.001	5.28	<0.001	***
Embeddedness (OP) of important tie*Earthquake intensity dummy	-0.0159	0.008	-1.94	0.052	·
Tie strength*Embeddedness (OP) of important tie*Earthquake intensity dummy	-0.0001	<0.001	-1.11	0.269	
Family plan size (1 to 5)	0.1902	0.012	16.25	<0.001	***
Degree centrality of ego	0.0006	<0.001	1.41	0.160	
Total call frequency of ego	-0.0019	<0.001	-21.05	<0.001	***
Total text frequency of ego	-0.0005	<0.001	-4.11	<0.001	***
Internet usage frequency of ego	<0.0001	<0.001	1.11	0.268	
Total usage frequency of other instant messaging of ego	0.0003	<0.001	1.34	0.179	
Smartphone dummy (1 = smartphone user)	-0.0519	0.020	-2.56	0.011	*
Roaming dummy (1 = traveling outside of prefecture)	0.2977	0.027	11.16	<0.001	***
Rural dummy (1 = rural)	0.0855	0.033	2.57	0.010	*
Damage dummy (1 = cell towers damaged)	0.3037	0.583	0.52	0.602	
Constant	-1.4550	0.353	-4.12	<0.001	***
Pseudo R squared: 0.2309					

Number of obs = 29,516

Probit model. Communications variables are average monthly data from 4 weeks before the earthquake. Fixed effects for 159 counties are included.

Table S20. Prediction accuracy of decision trees

Tie Strength	Embeddedness	Model 1	Model 2	Model 3
No	No	61.09%	85.99%	73.16%
Yes	No	61.18%	86.86%	77.92%
No	Yes	61.35%	86.89%	80.58%
Yes	Yes	62.13%	86.93%	85.80%

Note: We adopt a common data split ratio of 80% for model training and 20% for model testing.

Table S21. Prediction accuracy of tree models for Model 3

Tie Strength	Embeddedness	Single Tree	Random Forest (100 tress)
No	No	73.16%	73.16%
Yes	No	77.92%	78.63%
No	Yes	80.58%	80.97%
Yes	Yes	85.80%	85.87%

Note: We adopt a common data split ratio of 80% for model training and 20% for model testing.

Figure S12. Decision tree for activation latency (analogous to Model 1)

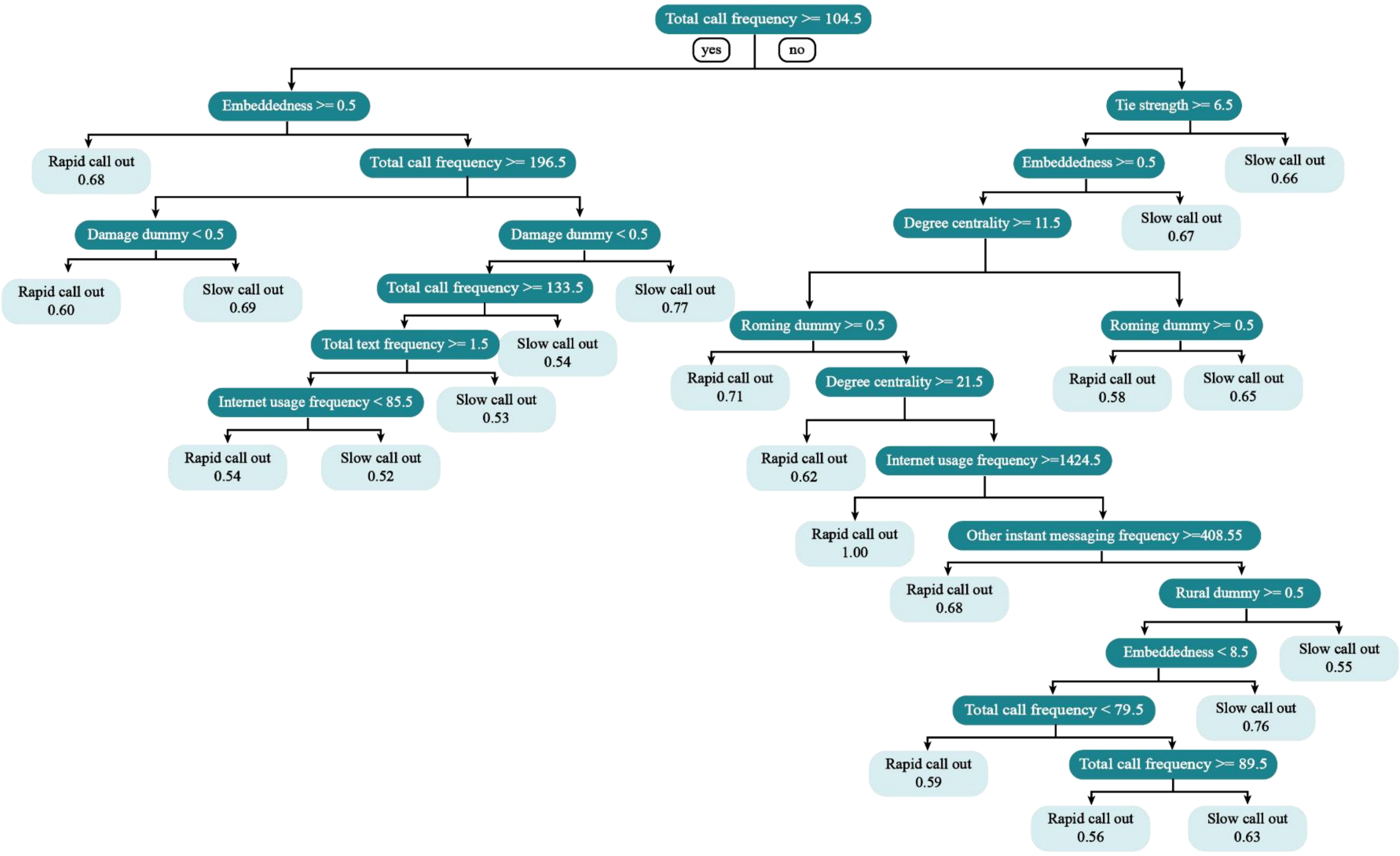

Figure S13. Decision tree for immediate reciprocity (analogous to Model 2)

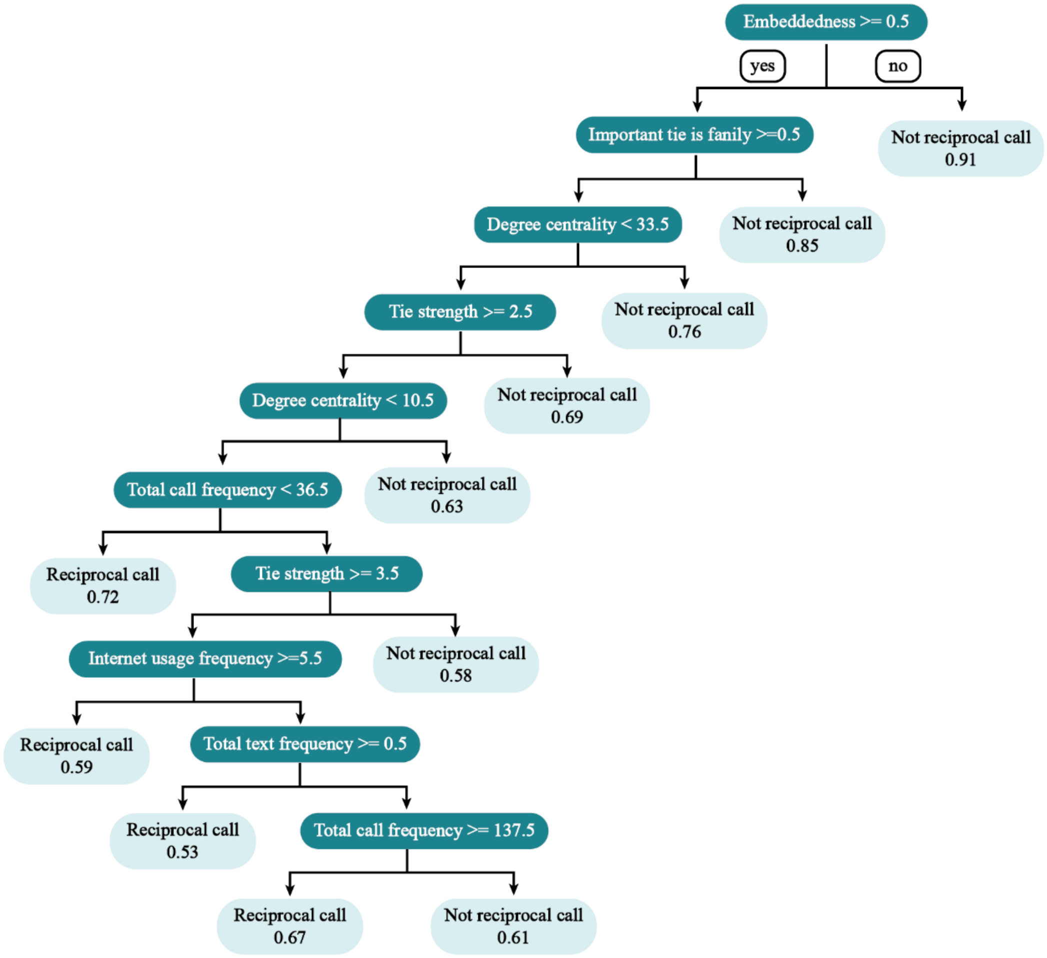

Figure S14. Decision tree for activation of family plan ties (analogous to Model 3)

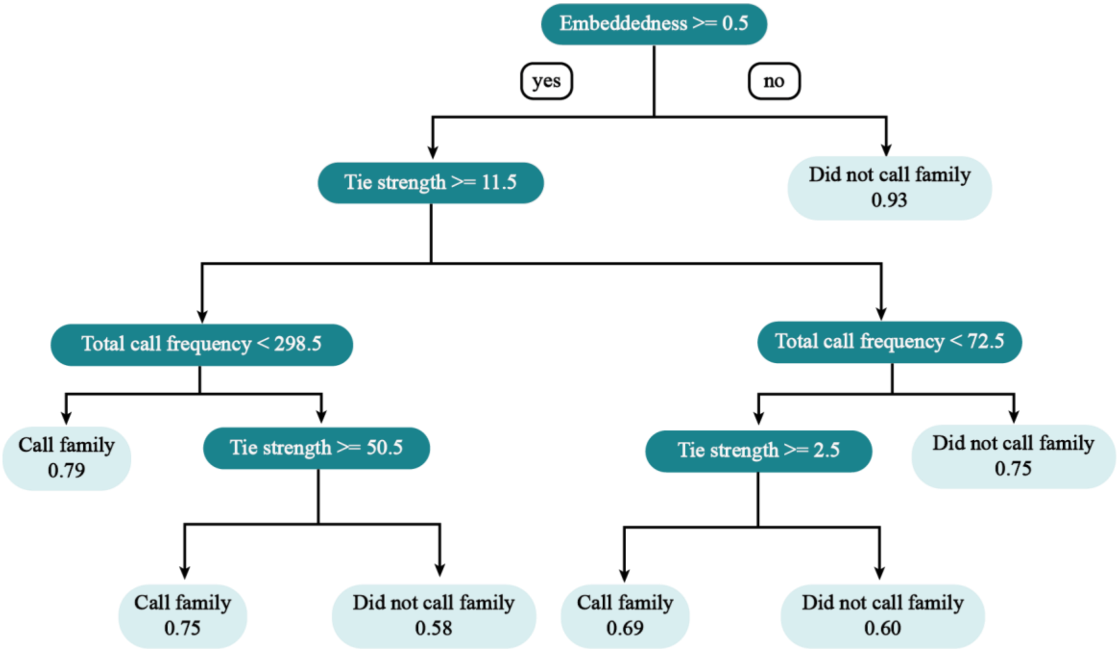

SI References

1. Ball B, Karrer B, Newman MEJ. 2011. Efficient and principled method for detecting communities in networks. *Phys. Rev.* E 84.
2. Barabási A-L. 2005. The origin of bursts and heavy tails in human dynamics. Nature 435:207–211.
3. Barbosa, H., Barthelemy, M. and Ghoshal, G. 2018. Human Mobility: Models and Applications. *Physics Reports*, 734:1-74.
4. Eagle N, Pentland A, Lazer D. 2009. Inferring friendship network structure using mobile phone data. *Proc. Natl. Acad. Sci. USA* 106(36):15274-15278.
5. Jo HH, Saramäki J, Dunbar RIM, Kaski K. 2014. Spatial patterns of close relationships across the lifespan. *Sci. Rep.* 4:6988.
6. Liben-Nowell D, Novak J, Kumar R, Raghavan P, Tomkins A. 2005. Geographic routing in social networks. *Proc. Natl Acad. Sci. USA* 102:11623–11628.
7. Moody J, White DR. 2013. Structural Cohesion and Embeddedness: A Hierarchical Concept of Social Groups. *Am. Sociol. Rev* 68:103-127.
8. Onnela JP et al. 2007. Structure and tie strengths in mobile communication networks. *Proc. Natl. Acad. Sci. USA* 104(18):7332-7336.
9. Palchykov V, Kaski K, Kertesz J, Barabasi A-L, Dunbar RIM. 2012. Sex differences in intimate relationships. *Sci. Rep.* 2:370.
10. Saramäkia J, Leichtb EA, Lópezb E, Roberts SGB, Reed-Tsochasb F, Dunbar RIM. 2014. Persistence of social signatures in human communication. *Proc. Natl. Acad. Sci. USA* 111(3):942–947.
